# Differentially Timed Extracellular Signals Synchronize Pacemaker Neuron Clocks

**DOI:** 10.1371/journal.pbio.1001959

**Published:** 2014-09-30

**Authors:** Ben Collins, Harris S. Kaplan, Matthieu Cavey, Katherine R. Lelito, Andrew H. Bahle, Zhonghua Zhu, Ann Marie Macara, Gregg Roman, Orie T. Shafer, Justin Blau

**Affiliations:** 1 Department of Biology, New York University, New York, New York, United States of America; 2 Department of Molecular, Cellular, and Developmental Biology, University of Michigan, Ann Arbor, Michigan, United States of America; 3 Department of Biology and Biochemistry, University of Houston, Houston, Texas, United States of America; 4 Center for Genomics & Systems Biology, New York University Abu Dhabi Institute, Abu Dhabi, United Arab Emirates; 5 Program in Biology, New York University Abu Dhabi, Abu Dhabi, United Arab Emirates; Washington University Medical School, United States of America

## Abstract

Synchronized neuronal activity is vital for complex processes like behavior. Circadian pacemaker neurons offer an unusual opportunity to study synchrony as their molecular clocks oscillate in phase over an extended timeframe (24 h). To identify where, when, and how synchronizing signals are perceived, we first studied the minimal clock neural circuit in *Drosophila* larvae, manipulating either the four master pacemaker neurons (LN_v_s) or two dorsal clock neurons (DN_1_s). Unexpectedly, we found that the PDF Receptor (PdfR) is required in both LN_v_s and DN_1_s to maintain synchronized LN_v_ clocks. We also found that glutamate is a second synchronizing signal that is released from DN_1_s and perceived in LN_v_s via the metabotropic glutamate receptor (mGluRA). Because simultaneously reducing *Pdfr* and *mGluRA* expression in LN_v_s severely dampened Timeless clock protein oscillations, we conclude that the master pacemaker LN_v_s require extracellular signals to function normally. These two synchronizing signals are released at opposite times of day and drive cAMP oscillations in LN_v_s. Finally we found that PdfR and mGluRA also help synchronize Timeless oscillations in adult s-LN_v_s. We propose that differentially timed signals that drive cAMP oscillations and synchronize pacemaker neurons in circadian neural circuits will be conserved across species.

## Abstract 2

Circadian pacemaker neurons in Drosophila are regulated by two synchronizing signals that are released at opposite times of day, generating a rhythm in intracellular cyclic AMP.

## Author Summary

Circadian molecular clocks are essential for daily cycles in animal behavior and we have a good understanding of how these clocks work in individual pacemaker neurons. However, the accuracy of these individual clocks is meaningless unless they are synchronized with one another. In this study we show that synchronizing the principal pacemaker LN_v_ neurons in *Drosophila* larvae require two extracellular signals that are received at opposite times of day: namely, the neuropeptide PDF released from LN_v_s themselves at dawn and glutamate released from dorsal clock neurons at dusk. LN_v_s perceive both PDF and glutamate via G-protein coupled receptors that increase or decrease intracellular cAMP, respectively. The alternating phases of PDF and glutamate release generate oscillations in intracellular cyclic AMP. In addition to maintaining synchrony between LN_v_s, this rhythm is also required for molecular clock oscillations in individual larval LN_v_s. We show that disruption of PDF and glutamate signaling also reduces synchrony in adult LN_v_s. This impairs the oscillations of clock proteins and flies have delayed onset of sleep. Our data highlight the importance of intercellular signaling in ensuring synchrony between clock neurons within the circadian network. Our findings help extend the conservation of clock properties between *Drosophila* and mammals beyond clock genes to include clock circuitry.

## Introduction

Coordinated neuronal activity is vital for neural networks to regulate complex processes such as behavior. Synchrony can be studied at the microsecond level by measuring neuronal activity, with synchronous activity often achieved via gap junctions that electrically couple neurons [1]. The circadian system offers an unusual opportunity to study synchrony over a much longer timeframe as circadian pacemaker neurons have molecular clocks that oscillate with 24 hour periods. These endogenous clocks drive daily rhythms in pacemaker neuron electrical activity and allow organisms to anticipate environmental transitions such as sunrise and sunset [2]. Although the molecular basis of the circadian clock is well established, how individual clock neurons remain synchronized is much less well understood. Synchrony is essential in the circadian system as the accuracy of individual clocks would be meaningless if they were desynchronized. Coordinated molecular clocks presumably ensure that an animal has a single internal representation of time.

In mammals, the primary circadian pacemaker in the suprachiasmatic nucleus (SCN) consists of ventral “core” and dorsal “shell” regions of clock neurons. Although SCN clock neurons exhibit 24 hour oscillations of clock proteins, anatomically distinct neurons oscillate with different phases (reviewed by [3]). Oscillations within different SCN neurons are coupled through cyclic AMP (cAMP) and Ca^2+^-dependent mechanisms, promoting synchrony and increasing the amplitude of individual oscillators compared to non-SCN clock neurons (reviewed by [4]). Synchronizing the different phases of SCN oscillations requires RGS16, which is rhythmically expressed and inactivates the G-protein Gαi to increase cAMP levels in the SCN in a time-dependent manner [5].

The *Drosophila* clock circuit also contains distinct groups of neurons including the small ventral Lateral Neurons (s-LN_v_s) that communicate with a subset of dorsal Lateral Neurons (LN_d_s) and Dorsal clock neurons (DNs) to generate bimodal locomotor activity rhythms in light∶dark (LD) cycles [6],[7]. s-LN_v_s are often called master pacemaker neurons as they set the period for most of the clock network in constant darkness (DD) [8]. However, robust behavioral rhythms in DD require LN_v_ and non-LN_v_ neurons to signal at different times of day [9]. Different groups of clock neurons also respond differently to environmental stimuli, such as day length or temperature [10],[11], leading to a network view of the clock where different clock neuron groups process information and communicate to keep time for an individual animal [12].

The mammalian neuropeptide VIP and the *Drosophila* neuropeptide PDF are found in subsets of clock neurons: ventral core SCN neurons in mice and LN_v_s in flies [3],[13]. VIP and PDF are both required for robust behavioral rhythms, the maintenance of stable phase relationships between different groups of clock neurons, and synchronized molecular clock oscillations within individual groups of clock neurons [8],[14]–[17]. The PDF receptor (PdfR) and VIP receptor VPAC2R are also required for robust rhythms of behavior, and they both activate Gαs to increase cAMP levels, indicating a conserved mode of action [18]–[21]. However, the precise mechanisms by which signaling across the clock circuit promotes synchronous clock oscillations remain unclear.

We used *Drosophila* to understand how circadian networks are synchronized, taking advantage of the exquisite precision with which individual groups of clock neurons can be manipulated in flies and the variety of genetic tools available. We made extensive use of the minimal larval clock circuit, which has only nine clock neurons per brain lobe, including four PDF-expressing LN_v_s that display synchronous clock protein oscillations in constant darkness (DD). These rhythms require the transcription factors Clock (CLK) and Cycle (CYC) that activate *period* (*per*) and *timeless* (*tim*) transcription. PER and TIM proteins dimerize, enter the nucleus, and then inhibit CLK/CYC activity. This represses expression of *per*, *tim*, and other CLK/CYC targets, including *vrille* and *Par Domain Protein 1* (*Pdp1*), that in turn feed back to regulate *Clk* expression (reviewed by [22]). One entire cycle takes 24 hours.

Synchronized LN_v_ oscillations in adult flies require PDF, as s-LN_v_ clocks become desynchronized in *Pdf^01^* null mutants after 6–9 days in constant darkness [17]. Here we show that LN_v_ synchrony in DD is a very active process, as desynchrony can be detected as early as 3 hours into the first subjective morning in *Pdf^01^* mutant larvae. We show that synchronized LN_v_ clocks require two distinct signals: a neuropeptide signal (PDF) received around dawn via PdfR and a neurotransmitter signal (glutamate) received from DN_1_s around dusk via the metabotropic glutamate receptor (mGluRA).

Surprisingly, simultaneously reducing expression of *Pdfr* and *mGluRA* in LN_v_s severely dampened TIM protein oscillations and blocked larval behavioral rhythms. Thus, oscillations of core clock proteins within pacemaker neurons require signals from other clock neurons. PdfR and mGluRA are GPCRs, and we show that daily oscillations in LN_v_ cAMP levels depend on their receiving PDF and glutamate. Because cAMP has previously been shown to be a molecular clock component in mammals [23], our data provide a mechanism for how extracellular signals impact molecular oscillations and neuronal synchrony. We extend these findings to adult flies and show that PdfR and mGluRA are required to maintain synchronized high-amplitude TIM oscillations in s-LN_v_s. In adults, desynchronized s-LN_v_ molecular clocks are associated with noisy behavioral rhythms, including delayed onset of sleep and increased nighttime activity.

Our data reveal a surprising degree of conservation in the mechanisms promoting synchronous clock oscillations in the mammalian SCN and *Drosophila* LN_v_s. This mirrors the conserved molecular basis of mammalian and *Drosophila* clocks and indicates that studying the simple *Drosophila* circadian neural circuit will help understand the more complex mammalian circadian system.

## Results

### PDF Signaling Synchronizes Larval LN_v_s

**Figure 1:**
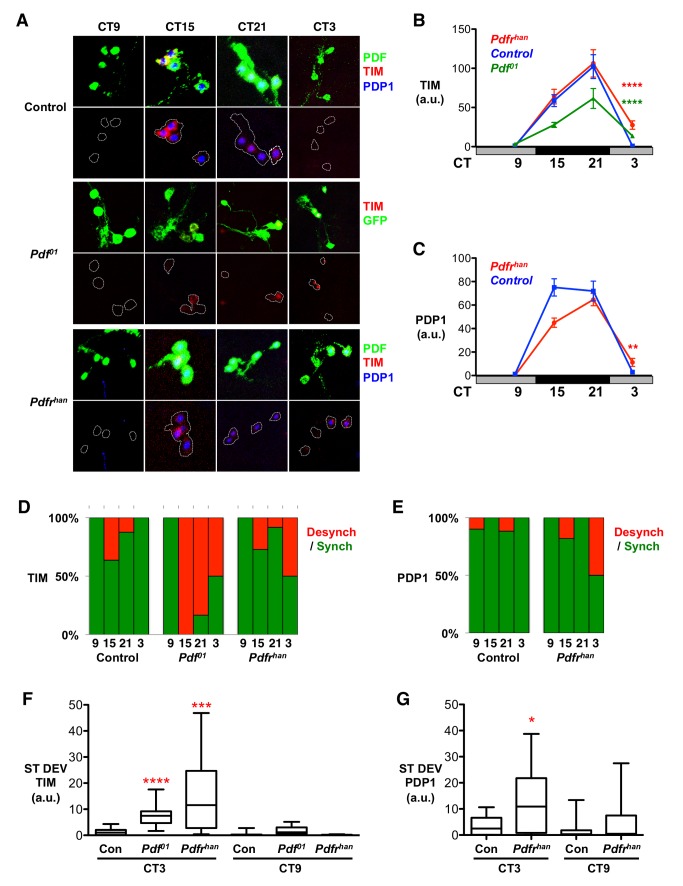
Synchronized TIM and PDP1 oscillations in LN_v_s depend on PDF signaling. Larval LN_v_s were immunostained using TIM, PDP1, and PDF antibodies at CT 9, 15, 21, and 3 on days 2–3 in DD after 4 days prior entrainment to 12∶12 LD cycles. Desynchrony data were calculated from 3–5 independent experiments, each with at least three brains. Error bars represent SEM. For total number of LN_v_ clusters analyzed, see Table S1. * *p*<0.05; ** *p*<0.01; *** *p*<0.001; **** *p*<0.0001. (A) Representative images of *y w* (Control, top panels), *Pdf^01^* mutants (middle), and *Pdfr^han^* mutants (bottom) stained for PDF or GFP (green), TIM (red), and PDP1 (blue). The lower panels for each genotype are the same images with the green channel removed and replaced by a dashed white line outlining the LN_v_s. *Pdf^01^* LN_v_s were identified via anti-GFP antibody staining of a UAS-GFP transgene driven by *Pdf-Gal4*, and PDP1 was not included in this experiment. (B) TIM immunostaining was quantified in Control (blue), *Pdfr^han^* (red), and *Pdf^01^* (green) LN_v_s on days 2 and 3 in DD. TIM oscillates in *Pdfr^han^* (ANOVA F_3,37_ = 13.68, *p*<0.0001) and *Pdf^01^* (ANOVA F_3,56_ = 16.80, *p*<0.0001) mutants. However, there is significantly more TIM at CT3 on day 3 in *Pdfr^han^* and *Pdf^01^* mutant LN_v_s than in control LN_v_s (Student's *t* test, both *p*<0.0001). At CT15, TIM levels are significantly reduced in *Pdf^01^* mutants compared to *Pdfr^han^* or control LN_v_s (Student's *t* test, both *p*<0.0003). (C) PDP1 immunostaining was quantified in LN_v_s of Control (blue) and *Pdfr^han^* mutant (red) larval brains on days 2 and 3 in DD. PDP1 oscillates in *Pdfr^han^* LN_v_s (ANOVA, F_3,37_ = 46.22, *p*<0.0001). PDP1 levels were significantly higher at CT3 on day 3 in *Pdfr^han^* mutant LN_v_s than in control LN_v_s (Student's *t* test, *p*<0.01). (D and E) Histograms show the percentage of LN_v_ clusters in which TIM (D) or PDP1 (E) was detected in either none or all four LN_v_s (“synchronized,” green bars) or in one, two, or three LN_v_s (“desynchronized,” red bars). (F and G) To further quantify desynchrony, the standard deviation (ST DEV) in TIM (F) or PDP1 (G) levels within a cluster of control, *Pdf^01^*, and *Pdfr^han^* mutant LN_v_s is shown as a box plot. Statistical comparisons by ANOVA with Tukey's post hoc test reveal significant increases in ST DEV in TIM in *Pdf^01^* (F_3,55_ = 26.71, *p*<0.0001) and *Pdfr^han^* (F_3,53_ = 12.13, *p*<0.0001) mutant LN_v_s compared to control LN_v_s at CT3 but not CT9. The ST DEV in PDP1 in *Pdfr^han^* mutant LN_v_s was also significantly elevated at CT3 but not CT9 (F_3,52_ = 5.03, *p* = 0.004). The box shows the 25th–75th percentile, and whiskers represent the 95% confidence interval.

The four PDF-expressing LN_v_s in each larval brain lobe are precursors of adult s-LN_v_s. Molecular clock oscillations in larval LN_v_s are normally tightly synchronized, oscillating in phase with each other so that TIM and PDP1 clock proteins are detectable in all four LN_v_s at CT21 and undetectable in all four LN_v_s 6 hours later at CT3 (Figure 1A) (CT, Circadian time, hours in constant darkness).

Because PER protein rhythms in adult s-LN_v_s become desynchronized in *Pdf^01^* null mutants in DD [17], we first tested whether PDF is required to synchronize larval LN_v_ molecular clocks. We measured TIM protein levels instead of PER with the rationale that TIM's shorter half-life [24],[25] would allow us to detect desymchrony earlier in DD.

To visualize LN_v_s in *Pdf^01^* mutants, we used the Gal4/UAS system [26] to express GFP in LN_v_s using the *Pdf-Gal4* driver. We measured TIM levels in LN_v_s isolated at CT9, CT15, and CT21 on the second day in DD and at CT3 on day 3. TIM continues to oscillate in *Pdf^01^* mutants, indicating that the molecular clocks in their LN_v_s are functional (Figure 1A–B). However, the amplitude of TIM rhythms in *Pdf^01^* mutants was reduced compared to controls (Figure 1B), as expected from the reduced amplitude *tim* RNA oscillations in *Pdf^01^* adult flies [27]. Closer inspection identified a mixture of TIM-positive and TIM-negative LN_v_s in a single brain lobe at CT15, 21, and 3 in *Pdf^01^* mutants (Figures 1D and S1A; see Materials and Methods), which we term desynchronized. Elevated desynchrony likely accounts for the significantly lower average TIM levels in *Pdf^01^* LN_v_s at CT15 and 21 than in controls (Figure 1B), in agreement with previous reports [17],[27].

We also quantified the variability within individual LN_v_ clusters by calculating the standard deviation in TIM levels across a single cluster. Figure 1F shows the distribution of standard deviations in TIM levels for each control or *Pdf^01^* LN_v_ cluster at CT3 and CT9. We chose CT3 because desynchronized LN_v_ clusters were only rarely found at this timepoint in control larvae. In contrast, TIM was detected in one, two, or three of the four LN_v_s in 50% of *Pdf^01^* LN_v_ clusters at CT3 (*n* = 20; Figures 1D and S1A and Table S1). In subsequent experiments we used the presence of TIM in a subset of LN_v_s at CT3 to indicate that an LN_v_ cluster had lost its normal coherent phase relationship and had become desynchronized, even if we did not observe desynchrony at other time points.

Our data show significantly more variability in TIM levels within an LN_v_ cluster in *Pdf^01^* mutants than in control larvae at CT3 (Figure 1F), reflecting desynchronized molecular clocks in *Pdf^01^* LN_v_s. No significant increase in standard deviation was observed in *Pdf^01^* mutants compared to controls at CT9 (Figure 1F). Indeed, the low TIM levels at CT9 indicate that *Pdf^01^* LN_v_ molecular clocks still oscillate as shown previously [17],[27].

Because PDF signals via PdfR, we next tested whether the synchrony of larval LN_v_ molecular clocks is also altered in *Pdfr* mutants. Although overall TIM oscillations were similar between control and *Pdfr^han5304^* (*Pdfr^han^*) hypomorphs, we observed higher TIM levels at CT3 in *Pdfr^han^* than in control larvae (Figure 1A–B). As with *Pdf^01^* null mutants, this is because TIM was detected in 1–3 of the four LN_v_s in 48% of *Pdfr^han^* mutant LN_v_ clusters at CT3 (Figures 1D and S1A). We found similar results for PDP1 (Figures 1A,C,E and S1B). In contrast, TIM or PDP1 expression was detected in <5% of control LN_v_s at CT3 (*n* = 21; Figure 1D–E and Table S1). The standard deviations in TIM and PDP1 levels are significantly elevated at CT3 in *Pdfr^han^* mutants compared to controls (Figure 1F–G). Thus PdfR, like PDF, is required for LN_v_s to stay synchronized.

In contrast to *Pdf^01^* LN_v_s, *Pdfr^han^* mutants did not show many desynchronized LN_v_ clusters at CT15 or CT21, and there was no corresponding reduction in the amplitude of TIM oscillations in *Pdfr^han^* mutants compared to control LN_v_s. This could be because *Pdfr^han^* is a hypomorph rather than a null allele and/or because type II GPCRs tend to be promiscuous, so receptors other than PdfR may also respond to PDF [28].

We also tested whether LN_v_ molecular clocks required PDF to maintain synchrony under LD cycles. We measured TIM and PDP1 levels in control larvae and in *Pdf^01^* and *Pdfr^han^* mutants at ZT3, but detected no TIM or PDP1 expression in LN_v_s (Figure S1C). Thus, light overrides desynchrony in *Pdf^01^* and *Pdfr^han5304^* mutants, with PDF signaling required for synchronous LN_v_ clock oscillations only in DD.

### PdfR Functions in Both LN_v_s and Other Clock Neurons to Synchronize LN_v_ Clocks

Because adult and larval LN_v_s express *Pdfr* ([29],[30] and Figure S2C), the simplest model to explain how PDF promotes LN_v_ synchrony would be that the four larval LN_v_s signal to synchronize each other via PDF and PdfR. However, *Pdfr* is also expressed in many non-LN_v_ adult clock neurons [29] and in larval DN_1_s (Figure S2A,B). Thus PDF signaling to non-LN_v_s could also be required for LN_v_ synchronization. We therefore used a *Pdfr^RNAi^* transgene [31] to reduce *Pdfr* levels in subsets of clock neurons to determine where PDF signaling is required for LN_v_ synchronization. Expressing *Pdfr^RNAi^* in LN_v_s significantly reduced the cAMP response of LN_v_s to PDF, indicating that *Pdfr^RNAi^* likely reduces *Pdfr* expression (Figure S2C). *UAS-Dicer-2* (*UAS-Dcr-2*) was co-expressed to increase RNAi efficacy in this and in all subsequent RNAi experiments unless otherwise stated, but is omitted from written genotypes in the text for simplicity.

**Figure 2:**
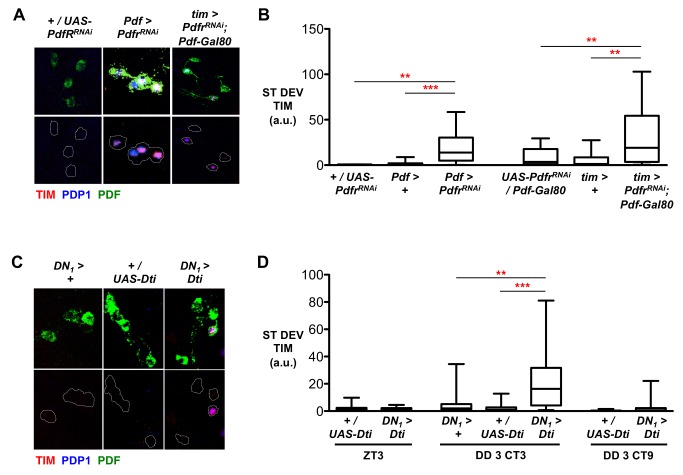
LN_v_ and non-LN_v_ clock neurons maintain LN_v_ synchrony. All experimental lines and *Pdf>*+control larvae in RNAi experiments include *UAS-Dcr-2*, but this is omitted from written genotypes for simplicity. Desynchrony data were calculated from 3–4 independent experiments, each consisting of at least three but usually five or more brains. Total number of LN_v_ clusters analyzed are in Table S1. ** *p*<0.01; *** *p*<0.001. (A) Representative images of LN_v_s in control larvae (*+*/*UAS-Pdfr^RNAi^*) or in larvae with reduced *Pdfr* levels in LN_v_s (*Pdf>Pdfr^RNAi^*) or all clock neurons except LN_v_s (*tim-Gal4; Pdf-Gal80*>*Pdfr^RNAi^*) immunostained for PDF (green), TIM (red), and PDP1 (blue) at CT3 on day 3 in DD. The lower panels for each genotype are the same images with the green channel (PDF) removed and replaced by a dashed white line outlining LN_v_s. (B) Box plots showing the ST DEV in TIM expression as in Figure 1. Statistical comparisons by ANOVA with Tukey's post hoc test show both *Pdf>Pdfr^RNAi^* (F_2,49_ = 12.33, *p*<0.0001) and *tim-Gal4; Pdf-Gal80*>*Pdfr^RNAi^* (F_2,51_ = 8.158, *p* = 0.0008) significantly increase the ST DEV of TIM levels compared to parental controls, reflecting increased desynchrony. (C) Representative images of larval LN_v_s stained for PDF (green), TIM (red), and PDP1 (blue) at CT3 on day 3 in DD. From left to right, Control *DN_1_>+*, and +/*UAS-Dti* LN_v_ clusters, and a representative desynchronized *DN_1_>Dti* LN_v_ cluster. The green channel (PDF) has been removed from the lower panel and replaced by a dashed white outline of LN_v_s. (D) Box plots showing quantification of desynchrony through measurement of ST DEV in TIM expression in larval LN_v_s in control or DN_1_ ablated larvae at ZT3, CT3, and CT9. *DN_1_>Dti* increases ST DEV at CT 3 compared to both parental controls (ANOVA with Tukey's post hoc test, F_2,49_ = 10.5, *p*<0.0001). There was no significant difference between *DN_1_>Dti* and controls at ZT3 (Student's *t* test, *p* = 0.35) or CT9 (Student's *t* test, *p* = 0.31).

We first targeted *Pdfr^RNAi^* to LN_v_s using *Pdf-Gal4* (denoted as *Pdf>*). At CT3 on day 3 of DD, TIM staining revealed that 44% of *Pdf>Pdfr^RNAi^* larvae had desynchronized LN_v_s, whereas >93% of control LN_v_s were synchronized (Figures 2A and S3A and Table S1). The standard deviation in TIM levels was also significantly increased in *Pdf>Pdfr^RNAi^* larvae compared to controls at CT3 (Figure 2B). Similar results were observed for PDP1 at CT3 (Figure S3B and Table S1).

Next, *Pdfr* expression was reduced in all non-LN_v_ clock neurons using the *tim-Gal4; Pdf-Gal80* driver combination (*tim; Pdf-Gal80>*). We found that 44% of LN_v_s were desynchronized in *tim*; *Pdf-Gal80>Pdfr^RNAi^* larvae (Figure S3A and Table S1). This probably underestimates the level of defective TIM oscillations, as 16% of *tim*; *Pdf-Gal80>Pdfr^RNAi^* LN_v_ clusters showed four LN_v_s expressing TIM at CT3, compared to only 6% of controls (Table S1). There is a corresponding increase in the standard deviation in TIM levels in *tim*; *Pdf-Gal80>Pdfr^RNAi^* LN_v_ clusters compared to control LN_v_s (Figure 2B). Similar results were observed for PDP1 (Figure S3A–B). These data indicate that LN_v_ synchrony depends on PdfR activity in both LN_v_ and non-LN_v_ clock neurons.

### DN_1_s Synchronize Molecular Clock Oscillations in LN_v_s

The non-LN_v_ clock neurons releasing the synchronizing signal could be the larval DN_1_s, the DN_2_s, or the fifth LN_v_. DN_1_s are the best candidates, as they project to LN_v_ axonal termini and modulate LN_v_ outputs by releasing glutamate to generate circadian rhythms in larval light avoidance [9]. Larval DN_1_s also respond directly to PDF (Figure S2A).

We therefore used *cry-Gal4* and *Pdf-Gal80* (*DN_1_>*) to target transgene expression exclusively to DN_1_s [9]. We first tested whether DN_1_ ablation affected LN_v_ synchrony by expressing Diptheria Toxin in DN_1_s (*DN_1_>Dti*). We found that TIM rhythms persisted in LN_v_s after DN_1_ ablation (Figure S3D), indicating that LN_v_s do not require DN_1_s for oscillations per se. However, TIM levels at CT3 on both days 2 and 3 in DD were elevated in DN_1_-ablated larvae (Figure S3D). Examining TIM staining in DN_1_-ablated brains in DD revealed that 50% of LN_v_ clusters were desynchronized at CT3 on days 2 and 3 in DD, a significant increase compared to controls (Figures 2C,D and S3A and Table S1). We observed similar increases in desynchrony of PDP1 expression when DN_1_s were ablated (Figures S3A,C,E and S6C–D and Table S1) with significantly higher levels at CT3 on day 3. We did not observe desynchrony in LD cycles (Figure 2D) or at CT9, just like *Pdf^01^* and *Pdfr^han^* mutants. We conclude that PDF signaling (Figure 1) and DN_1_s (Figure 2) normally maintain larval LN_v_ molecular clock synchrony in constant darkness.

### DN_1_ Glutamate Synchronizes LN_v_s

To test this model further, we sought to identify the DN_1_ signal and the relevant receptor in LN_v_s. Because larval DN_1_s are glutamatergic [32], we tested whether reducing DN_1_ glutamate levels alters LN_v_ molecular clock synchrony. *Glutamate decarboxylase 1* (*Gad1*) was mis-expressed in DN_1_s, to convert glutamate into GABA [9],[33], which cannot be released as DN_1_s do not produce the vesicular GABA transporter. Thus misexpression of *Gad1* reduces presynaptic glutamate. This manipulation does not affect DN_1_ viability, and their molecular clocks still oscillate [9].

**Figure 3:**
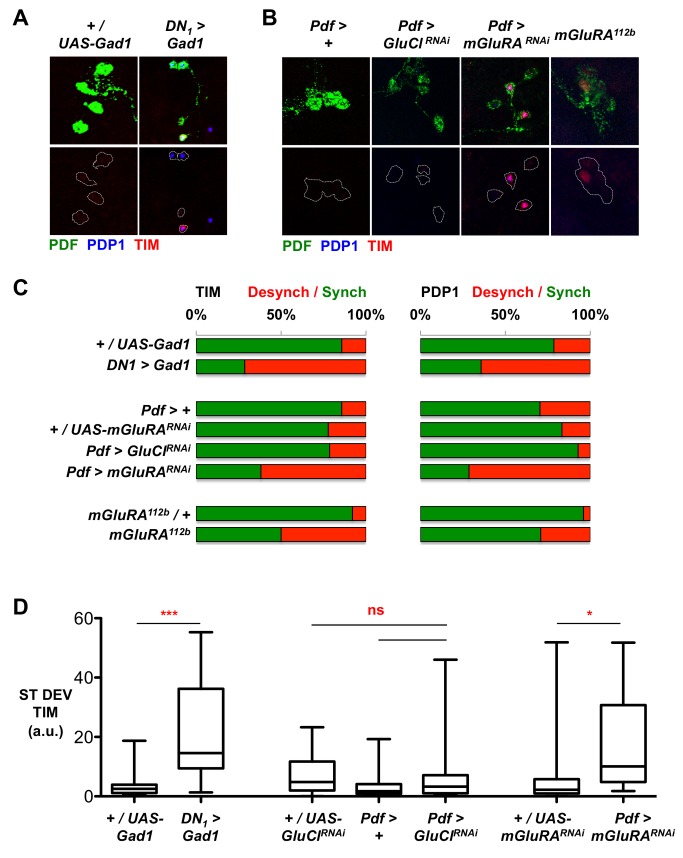
A DN_1_ glutamate signal mediated via mGluRA synchronizes LN molecular oscillations. All experimental lines and *Pdf>+*control larvae in RNAi experiments include *UAS-Dcr-2*, but this is omitted from written genotypes for simplicity. Desynchrony data were calculated from 2–5 independent experiments, each consisting of at least four brains. Total numbers of LN_v_ clusters analyzed are in Table S1. * *p*<0.05; *** *p*<0.001. (A and B) Representative images of larval LN_v_s stained for PDF (green), TIM (red), and PDP1 (blue) at CT3 on day 3 in DD. Genotypes in (A) are control(*+/UAS-Gad1*) and *DN_1_>Gad1* experimental larvae. Genotypes in (B) are control (*Pdf>+*) and experimental larvae in which *GluCl* (*Pdf>GluCl^RNAi^*) or *mGluRA* (*Pdf>mGluRA^RNAi^*) levels are reduced in LN_v_s, and *mGluRA^112b^/+* heterozygous control or *mGluRA^112b^* mutant LN_v_s. (C) Histograms showing percentage of synchronized (green) or desynchronized (red) LN_v_ clusters for TIM (left panel) or PDP1 (right panel) at CT3. Top: 14% of control (*+/UAS-Gad1*) LN_v_ clusters are desynchronized compared to 71% of *DN_1_>Gad1* LN_v_ clusters by TIM staining, and 21% of control (*+/UAS-Gad1*) LN_v_ clusters have detectable PDP1 expression compared to 64% in *DN_1_>Gad1* brains. Middle: ∼20% of *Pdf>GluCl^RNAi^* or +/*UAS-mGluRA^RNAi^* larval brains have desynchronized TIM levels compared to 62% of *Pdf>mGluRA^RNAi^* brains. Less than 20% of *Pdf>GluCl^RNAi^* or *+/UAS-mGluRA^RNAi^* larval brains have detectable PDP1 expression, compared to 71% of *Pdf>mGluRA^RNAi^* brains. Bottom: 50% of *mGluRA^112b^* mutant LN_v_s show desynchronized TIM expression, compared to 8% of *mGluRA^112b^/*+ controls. For PDP1, 29% of LN_v_ clusters are desynchronized in *mGluRA^112b^* mutants, compared to 4% of *mGluRA^112b^/*+ controls. In addition, 3/24 *mGluRA^112b^* mutants had all four LN_v_s expressing PDP1 compared to 0/25 control LN_v_ clusters. (D) Box plots showing quantification of desynchrony by measuring ST DEV in TIM levels within a cluster in larval LN_v_s in control, *DN_1_>Gad1*, *Pdf>GluCl^RNAi^*, and *Pdf>mGluRA^RNAi^* larvae at CT3 on day 3 in DD. *DN_1_>Gad1* (Student's *t* test, *p* = 0.0004) and *Pdf>mGluRA^RNAi^* (ANOVA with Tukey's post hoc test, F_2,50_ = 5.597, *p* = 0.0064) significantly increase the ST DEV in TIM levels, reflecting increased LN_v_ desynchrony, whereas *Pdf>GluCl^RNAi^* does not (ANOVA with Tukey's post hoc test, F_2,39_ = 0.93, *p* = 0.40).

We found that overall TIM oscillations were relatively normal in *DN_1_>Gad1* LN_v_s (Figure S4A). However, TIM levels were significantly elevated at CT3 in *DN_1_>Gad1* larvae (Figure S4A). This is because *DN_1_>Gad1* significantly increased LN_v_ desynchrony, determined by comparing the standard deviation in TIM and PDP1 expression with control LN_v_s (Figures 3A,C,D and S4C and Table S1). Therefore, we conclude that DN_1_s release glutamate to synchronize LN_v_ molecular clocks.

### LN_v_s Perceive the Synchronizing Glutamate Signal Via *mGluRA*

Larval LN_v_s express two glutamate receptors: a metabotropic glutamate receptor (mGluRA, [32]) and a glutamate-gated Chloride channel (GluCl, [9]). To determine whether one of these receptors transduces the glutamate signal to synchronize LN_v_s, we used RNAi transgenes previously shown to reduce expression of *mGluRA* or *GluCl* [9],[32]. We found that reducing *GluCl* expression in LN_v_s had no effect on TIM and PDP1 oscillations or LN_v_ synchrony (Figures 3B–D and S4B–C). In contrast, expressing *mGluRA^RNAi^* in LN_v_s produced similar molecular phenotypes to DN_1_ ablation, with elevated TIM levels at CT3 and 75% of LN_v_s desynchronized (Figure 3B–D and Table S1).

As an independent way to manipulate *mGluRA* expression, we measured TIM levels at CT3 in LN_v_s of *mGluRA^112b^* null mutant larvae (Figures 3B–C and S4D and Table S1). We found desynchronized LN_v_s in homozygous *mGluRA^112b^* mutant larvae but not in heterozygous controls. We saw similar levels of desynchronization when measuring PDP1 levels in *Pdf>mGluRA^RNAi^* and *mGluRA^112b^* mutant LN_v_s (Figures 3C and S4C–D). Taking these data together with our manipulations of DN_1_ glutamate levels, we conclude that glutamate released by DN_1_s helps synchronize LN_v_ oscillations via *mGluRA*.

We previously showed that LN_v_s require GluCl rather than mGluRA for circadian rhythms in the rapid light avoidance of larvae [9]. Thus, a single neurotransmitter, glutamate, released by DN_1_s has two distinct functions depending on the receptor in LN_v_s that perceives the signal. Presumably the rapid action of the ionotropic receptor on LN_v_ excitability [9] is best suited to regulate light avoidance behavior, whereas mGluRA acts on a slower timescale to regulate clock oscillations.

### PdfR and mGluRA Cooperate to Maintain LN_v_ Synchrony and Promote Strong TIM Oscillations

LN_v_s require two different signals to maintain synchrony, as reducing expression of either *Pdfr* or *mGluRA* desynchronized LN_v_ molecular clocks. However, we only observed an increase in desynchronized LN_v_ clusters at CT3 in *Pdf>Pdfr^RNAi^* or *Pdf>mGluRA^RNAi^* larval brains compared to controls, with most LN_v_ clusters remaining synchronized at CT21. This suggested that the second signal—glutamate in *Pdf>Pdfr^RNAi^* and PDF in *Pdf>mGluRA^RNAi^* larvae—maintains some degree of LN_v_ synchrony and we hypothesized that simultaneously reducing *Pdfr* and *mGluRA* expression would more strongly affect LN_v_ clock synchrony.

**Figure 4:**
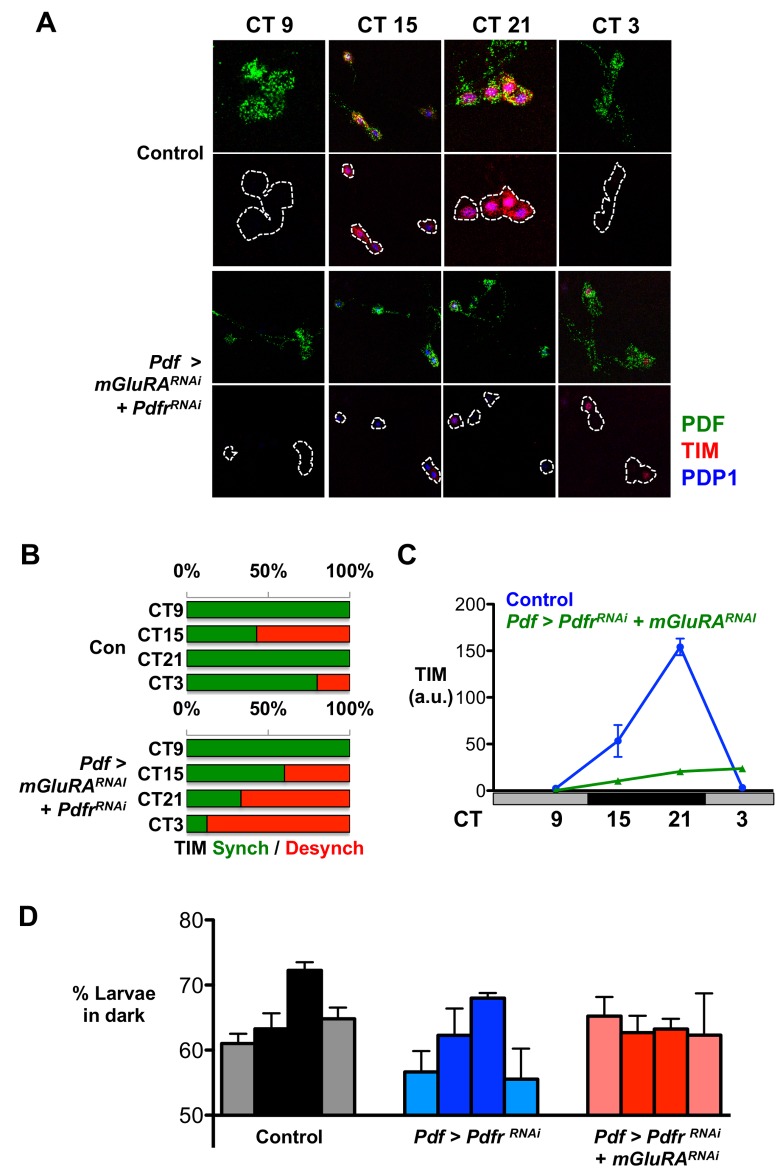
PdfR and mGluRA promote high-amplitude TIM oscillations and larval behavioral rhythms. All experimental lines and *Pdf>+*control larvae also include *UAS-Dcr-2* for RNAi experiments, but this is omitted from written genotypes for simplicity. Desynchrony data were calculated from 2–4 independent experiments, each consisting of at least five brains. Total number of LN_v_ clusters analyzed are in Table S1. Error bars represent SEM. (A) Representative images of larval LN_v_s at CT 9, 15, 21, and 3 on days 2–3 in DD for control (*+/UAS-mGluRA^RNAI^*; +/*UAS-Pdfr^RNAi^*) or *Pdf>mGluRA^RNAi^+Pdfr^RNAi^* larval LN_v_s immunostained for TIM (red), PDP1 (blue), and PDF (green). PDF staining is removed from lower panels, with LN_v_s indicated by a white line. (B) Histogram showing the number of synchronized (green) or desynchronized (red) LN_v_ clusters in control (*+/UAS-mGluRA^RNAI^*; +/*UAS-Pdfr^RNAi^*) or *Pdf>mGluRA^RNAi^+Pdfr^RNAi^* larval brains, determined by TIM staining at CT3. (C) Average TIM levels of control (blue) and *Pdf>mGluRA^RNAi^+Pdfr^RNAi^* (green) LN_v_s. TIM oscillations are dampened in *Pdf>mGluRA^RNAi^+Pdfr^RNAi^* larval LN_v_s (two-way ANOVA significant Genotype effect, F_1,102_ = 119.53, *p*<0.0001, and Genotype×Time interaction, F_3,102_ = 100.11, *p*<0.0001). (D) Larval light avoidance was measured by counting the number of larvae on the dark side of a Petri dish after 15 min. Light avoidance was assayed on day 2 (CT12, 18, 24) or day 3 (CT6) of DD after prior LD entrainment. Control (*Pdf>+*) larvae (grey) and *Pdf>Pdfr^RNAi^* larvae (blue) show similarly phased light avoidance rhythms, peaking at dawn (two-way ANOVA, no Genotype×Time interaction, F_3,22_ = 0.31, *p* = 0.82). *Pdf>mGluRA^RNAi^+Pdfr^RNAi^* larvae lose light avoidance rhythms (ANOVA F = 0.13, *p* = 0.94).

We measured TIM and PDP1 oscillations in LN_v_s expressing transgenes targeting both *Pdfr* and *mGluRA* expression (*Pdf>Pdfr^RNAi^+mGluRA^RNAi^*). We found that 88% of LN_v_ clusters showed desynchrony in TIM protein levels at CT3 (Figures 4A–B and S5B and Table S1) and 75% for PDP1 (Figure S5A,C and Table S1). *Pdf>Pdfr^RNAi^+mGluRA^RNAi^* larvae also had significantly more desynchronized LN_v_ clusters at CT21 and CT3 than control larvae (Figure S5A). Thus simultaneously reducing expression of both receptors dramatically increased the percentage of desynchronized LN_v_s compared to reducing *Pdfr* or *mGluRA* expression alone, indicating that PDF and glutamate signals work together to promote synchrony.

Although we observed a few individual LN_v_s with high TIM levels in *Pdf>Pdfr^RNAi^+mGluRA^RNAi^* larvae, overall TIM oscillations were almost completely lost (Figure 4C). This contrasts with the robust TIM oscillations of *Pdf>Pdfr^RNAi^* and *Pdf>mGluRA^RNAi^* single knock-down larvae (Figure S5E). High-amplitude TIM protein oscillations in LN_v_s thus depend on external signals, including PDF and glutamate, and are not fully cell-autonomous. Although PDP1 showed elevated desynchrony in *Pdf>Pdfr^RNAi^+mGluRA^RNAi^* LN_v_s (Figure S5A,C), overall PDP1 oscillations were relatively unaffected (Figure S5D). Thus *Pdf>Pdfr^RNAi^+mGluRA^RNAi^* LN_v_s are still partly functional. These data suggest that TIM is a more direct target than PDP1 in LN_v_s for the signaling pathways that transduce glutamate and PDF signals.

Do the reduced amplitude TIM rhythms in *Pdf>Pdfr^RNAi^+mGluRA^RNAi^* double mutant larvae affect behavioral rhythms? We had previously found that light avoidance rhythms require glutamate release from DN_1_s and transduction via GluCl in LN_v_s [9]. Because TIM oscillations in LN_v_s remained intact in *Pdf>GluCl^RNAi^* larval brains (Figure S4B), we concluded that glutamate received by GluCl modulates LN_v_ outputs rather than LN_v_ molecular clocks [9]. Knocking down either *mGluRA* or *Pdfr* individually in LN_v_s does not block TIM or PDP1 protein oscillations (Figure S5E–F) and larval light avoidance is still rhythmic, with peak levels at dawn (Figure 4D and [9]). However, we found that larvae with *mGluRA* and *Pdfr* expression simultaneously reduced in LN_v_s lose light avoidance rhythms (Figure 4D). This result suggests that TIM oscillations in LN_v_s are essential for light avoidance rhythms and that PDP1 rhythms alone cannot support larval rhythms. Overall, these data indicate the importance of extracellular signals for LN_v_s to oscillate normally and promote rhythmic behavior.

### mGluRA and PdfR Are Activated at Different Times of Day in LN_v_s

Adult s-LN_v_s are most excitable at dawn [34],[35] and drive the morning peak of locomotor activity [6],[7]. We previously showed that the same is likely true for the larval LN_v_s that control the dawn peak in light avoidance, whereas larval DN_1_s most likely signal at dusk [9]. To test whether DN_1_s signal at dawn or dusk to promote LN_v_ synchrony, we used a temperature-sensitive *Shibire* transgene (*UAS-Shi^ts^* [36]) to temporally block synaptic transmission.

**Figure 5:**
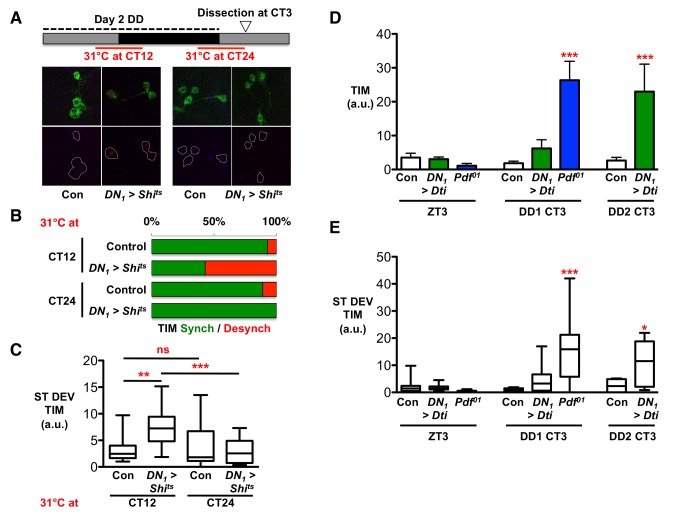
PDF and glutamate signal at different times of day to regulate LN_v_ cAMP levels. Statistical comparisons are by ANOVA with Tukey's post hoc test, unless otherwise stated. Desynchrony data were calculated from three independent experiments, each consisting of at least three brains. Total number of LN_v_ clusters analyzed are in Table S1. Error bars show SEM. Whiskers represent 95% confidence. * *p*<0.05; ** *p*<0.01; *** *p*<0.005. (A–C) Larvae were subjected to a heat pulse (6 hours at 31°C) from either CT9 to CT15 on day 2 (CT12 shift) or from CT21 on day 2 to CT3 on day 3 of DD (CT24 shift). Larvae were then dissected at CT3 on day 3 of DD and immunostained with αTIM (red), αPDP1 (blue), and αPDF (green). (A) Representative images of control (+/*UAS-Shi^ts^*) LN_v_s or LN_v_s of larvae expressing the temperature-sensitive allele of *Shibire* in DN_1_s (*DN_1_>Shi^ts^*). At 31°C, *Shi^ts^* is inactive, blocking synaptic transmission. Left: Effect of heat pulse at CT12. Right: Effect of heat pulse at CT24/0. (B) Histograms showing the percentage of LN_v_ clusters where TIM was detected in either none or all four of the four LN_v_s (“synchronized,” green bars) or in one, two, or three LN_v_s (desynchronized, red bars). (C) Desynchrony was quantified as in Figure 1 by measuring ST DEV in TIM expression. A CT12 heat pulse significantly increased ST DEV of TIM expression in *DN_1_>Shi^ts^* brains compared to controls and to *DN_1_>Shi^ts^* larval brains with a CT24 heat pulse (F_3,60_ = 6.423, *p* = 0.0008). (D) Larval LN_v_s were immunostained for TIM at ZT3 and CT3 on days 1 and 2 of DD in Control (*+/UAS-Dti*), *DN_1_>Dti*, and *Pdf^01^* mutants. DN_1_ ablation and *Pdf^01^* mutants do not affect LN_v_ TIM levels at ZT3 (F_3,41_ = 1.53, *p* = 0.22). On the first day of DD, only *Pdf^01^* increases TIM expression in LN_v_s (F_3,51_ = 11.43, *p*<0.0001). *DN_1_>Dti* increases TIM levels in LN_v_s on day 2 in DD (Student's *t* test, *p* = 0.0004). (E) Desynchrony of LN_v_s in LD and on days 1 and 2 of DD was quantified by measuring ST DEV of TIM expression in Control (*+/UAS-Dti*), *DN_1_>Dti*, and *Pdf^01^* mutants. The STDEV in TIM is significantly higher in *Pdf^01^* LN_v_s compared to control or *DN_1_>Dti* LN_v_s on the first day of DD, reflecting increased desynchrony (F_2,38_ = 16.48, *p*<0.0001). *DN_1_>Dti* increases desynchrony as measured by TIM ST DEV only on day 2 in DD (Student's *t* test, *p* = 0.019).

*Shi^ts^* was expressed specifically in DN_1_s (*DN_1_>Shi^ts^*), and larvae were maintained at the permissive temperature of 25°C for 4 days in LD and 1 day in DD. On the second day in DD, the temperature was elevated to the nonpermissive temperature of 31°C for 6 hours from either CT9 to CT15 (“CT12 shift”) or CT21 to CT3 (“CT24 shift”) to block DN_1_ signaling around dusk or dawn, respectively (Figure 5A). Larval brains were dissected at CT3 on day 3 of DD (i.e., 12 hours after the end of a CT12 temperature shift or immediately after the end of a CT24 temperature shift). We found that 57% of LN_v_ clusters showed desynchronized TIM levels when DN_1_ synaptic transmission was blocked at dusk (*DN_1_>Shi^ts^*, 31°C at CT12) compared to 7% of control LN_v_s (*UAS-Shi^ts^*/+; Figure 5A–C and Table S1). Similarly, 36% of LN_v_s in *DN_1_>Shi^ts^* larvae shifted to 31°C at CT12 had desynchronized PDP1 levels compared to 0% of control LN_v_s (Figure S6A–B and Table S1). In contrast, blocking synaptic transmission from DN_1_s around dawn had no effect on LN_v_ synchrony (*DN_1_>Shi^ts^*, 0% desynchronized for TIM or PDP1 with a CT24 heat pulse; Figures 5A–C and S6A–B and Table S1). We therefore conclude that DN_1_ signaling around dusk is required to synchronize LN_v_s.

To further test the idea that PDF and glutamate promote synchrony at different times of day, we took advantage of the synchronizing effect of LD cycles on *Pdf^01^* and *DN_1_>Dti* LN_v_s (Figures 2D, S1C, and S3B and Table S1). Based on the likely timing of LN_v_ and DN_1_ signals, wild-type LN_v_ clocks should have received the PDF signal at subjective dawn by CT3 on day 1 in DD, but not yet received the glutamatergic signal at subjective dusk. Thus we predicted that *Pdf^01^* mutants would show desynchrony at this time point, whereas *DN_1_>Dti* LN_v_s, which still receive the PDF signal, would not.

We measured TIM and PDP1 levels in LN_v_s at CT3 on the first day of DD and found higher TIM and PDP1 levels and an increase in the variability of clock protein levels between LN_v_s in the same cluster in *Pdf^01^* mutants, indicating that LN_v_s are already desynchronized just 3 hours into DD (Figures 5D–E and S6C–D). In contrast, the LN_v_ clocks in larvae with DN_1_s ablated remained synchronized at CT3 on the first day in DD, and desynchrony was first detected on day 2 (Figures 5D–E and S6C–D).

We interpret these data to mean that desynchrony in DN_1_-ablated larvae requires larvae to traverse subjective dusk when the DN_1_ signal is released. Because desynchrony appears on different days in *Pdf^01^* and DN_1_-ablated larvae, this supports the model where LN_v_ synchrony depends on PDF received at dawn and glutamate received at dusk. This is consistent with the previously reported timing of LN_v_ excitability [34],[35] and of the larval LN_v_ and DN_1_ signals that regulate light avoidance [9].

### LN_v_ cAMP Rhythms Require mGluRA and PdfR

PdfR and mGluRA are both G-protein coupled receptors. PdfR signals via Gαs [18],[19],[21],[37] and mGluRA can also alter cAMP levels [38]. Because cAMP is a clock component in mammals [23] and likely also in flies [39],[40], regulation of LN_v_ cAMP levels by extracellular signals could maintain LN_v_ synchrony and promote robust TIM oscillations.

**Figure 6:**
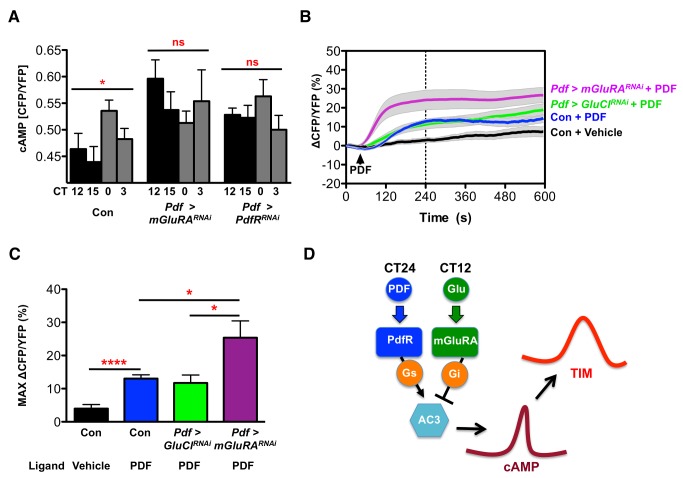
mGluRA and PdfR regulate intracellular cAMP. Statistical comparisons are by ANOVA with Tukey's post hoc test. Error bars show SEM. Whiskers represent 95% confidence. * *p*<0.05; ** *p*<0.01; *** *p*<0.001; **** *p*<0.0001. (A) Larvae were dissected and analyzed on day 2 in DD. CFP and YFP levels were measured in the projections of *Pdf>Epac1-camps* LN_v_s. The ratio of CFP/YFP reflects the basal level of cAMP. The CFP/YFP ratio oscillates in control (*Pdf>Epac1-camps*) LN_v_ projections, peaking at CT24 (ANOVA F_3,62_ = 2.933, *p* = 0.04). There is no significant oscillation in *Pdf>Epac1-camps*+*mGluRA^RNAi^* (F_3,59_ = 0.815, *p* = 0.49) or *Pdf>Epac1-camps*+*Pdfr^RNAi^* (F_3,47_ = 1.068, *p* = 0.37). The CFP/YFP ratio is significantly increased at CT12 in *Pdf>Epac1-camps*+*mGluRA^RNAi^* compared to control LN_v_s (F_2,38_ = 5.021, *p* = 0.0017) but not in *Pdf>Epac1-camps*+*Pdfr^RNAi^*, consistent with glutamate signals inhibiting cAMP at CT12. (B) Averaged Epac-1-camps CFP/YFP ratio responses to bath application of 100 nM PDF or vehicle (arrow). The wild-type (*Pdf>Epac1-camps*) response to 100 nM PDF is shown in blue, and the wild-type response to vehicle is shown in black. Knockdown of *GluCl* (*Pdf>Epac1-camps*+*GluCl^RNAi^*, green) had no significant effect on the response to PDF, but knockdown of *mGluRA* (*Epac1-camps*+*mGluRA^RNAi^*, magenta) significantly increased the cAMP response of LN_v_s to PDF. Vehicle traces represent 10 LN_v_ cell bodies from five brains (10, 5), wild-type PDF (10, 5), *Pdf>GluCl^RNAi^* PDF (20, 9), and *Pdf>mGluRA^RNAi^* PDF (27, 12). (C) Comparison of mean maximum Epac-1-camps CFP/YFP ratio changes between 0 and 240 s [dashed line in (B)] [genotypes and sample sizes as in (B)]. Application of 100 nM PDF significantly increased cAMP in LN_v_s of *Pdf>Epac1-camps* flies compared to vehicle (*p*<0.0001 by unpaired *t* tests). PDF responses of *Pdf>Epac1-camps*+*GluCl^RNAi^* LN_v_s were not significantly different from wild-type LN_v_s (*p* = 0.6217). PDF responses of *Pdf>Epac1-camps*+*mGluRA^RNAi^* LN_v_s were significantly higher than wild-type (*p* = 0.024) and *Pdf>Epac1-camps*+*GluCl^RNAi^* (*p* = 0.0193) LN_v_s. (D) Model: We propose that LN_v_s signal to each other via PDF around dawn. This signal is received by PdfR, which acts via Gαs/AC3 to increase intracellular cAMP. DN_1_s release glutamate around dusk. This signal is received by mGluRA in LN_v_s, which acts via Gαi to inhibit AC3 and reduce intracellular cAMP. Daily regulation of cAMP by external signals promotes robust TIM oscillations and LN_v_ synchrony.

We used the FRET-based Epac1-camps sensor [19] to measure basal cAMP levels on day 2 in DD. We first assayed control LN_v_s, focusing on their axonal termini near DN_1_ projections [9]. We found that cAMP levels, measured by the ratio of CFP/YFP, were highest at CT24, indicating that cAMP levels normally oscillate in LN_v_ projections (Figure 6A). Strikingly, cAMP (CFP/YFP) oscillations were lost in the projections of both *Pdf>Pdfr^RNAi^* and *Pdf>mGluRA^RNAi^* larval LN_v_s (Figure 6A).

We noticed that *Pdf>mGluRA^RNAi^* LN_v_ cAMP levels were significantly higher than controls at dusk (CT12), when DN_1_s signal for synchrony. This is consistent with data showing that mGluRA reduces cAMP levels by signaling via Gαi [38], thereby opposing PdfR activity [18],[37]. To test this idea, we measured the responsiveness of LN_v_s to PDF with reduced mGluRA activity. We first generated a PDF response curve to determine the minimal PDF concentration that elicits an Epac1-camps response (Figure S7A–C). We then tested whether expressing *mGluRA^RNAi^* in *Pdf>Epac1-camps* LN_v_s altered this response (Figure 6B–C) using *GluCl^RNAi^* as a control. We found that *mGluRA^RNAi^*, but not *GluCl^RNAi^*, significantly increased LN_v_ responsiveness to PDF (Figure 6C). Therefore, we propose that mGluRA acts in an opposite manner to PdfR and reduces intracellular cAMP.

To test if cAMP links to synchronized clock protein oscillations, we built on the recent identification of Adenylate cyclase 3 (AC3) as the specific Adenylate cyclase downstream of PdfR in LN_v_s [21]. We tested whether AC3 is required for LN_v_ synchronization by reducing expression of AC3 using two independent RNAi lines (*Pdf>AC3^TRiP RNAi^* and *Pdf>AC3^Vienna RNAi^*) that reduce PDF responses in LN_v_s [21]. We found that expressing each RNAi line in LN_v_s desynchronized TIM expression in 35%–40% of LN_v_ clusters and PDP1 expression in 28%–35% of LN_v_ clusters (Figure S8A–B and Table S1). Reducing *AC3* expression in LN_v_s also significantly increased desynchrony measured by standard deviation in TIM and PDP1 expression (Figure S8C–D).

We conclude that PdfR and mGluRA regulate LN_v_ cAMP levels at different times of day, presumably by regulating AC3 activity. This leads to a model in which LN_v_ cAMP rhythms are generated by extracellular signals, with PDF/PdfR increasing cAMP via AC3 around dawn, whereas glutamate inhibits the response of LN_v_s to PDF via mGluRA by inhibiting AC3 around dusk (Figure 6D). cAMP oscillations then feed into the molecular clock, affecting TIM oscillations through an unknown mechanism, which will be a topic of future research.

### PdfR and mGluRA Promote Molecular Clock Synchrony in Adult s-LN_v_s

**Figure 7:**
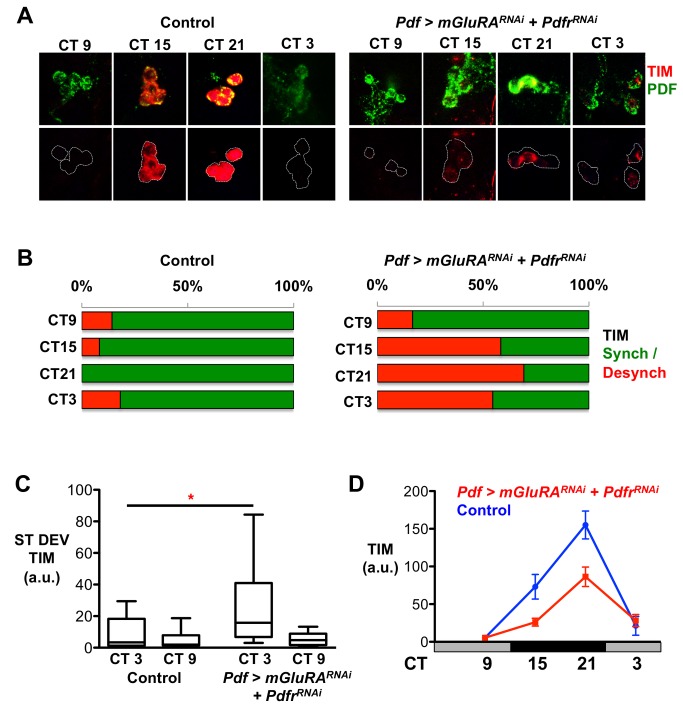
*mGluRA* and *PdfR* help synchronize molecular oscillations in adult s-LN_v_s. Experimental lines include *UAS-Dcr-2* for RNAi experiments, but this is omitted from written genotypes for simplicity. Desynchrony data were calculated from 2–3 independent experiments, each consisting of at least five brains. Total number of LN_v_ clusters analyzed are in Table S1. Whiskers represent 95% confidence interval. * *p*<0.05. (A) Images of Control (*+/UAS-mGluRA^RNAI^*; +/*UAS-Pdfr^RNAi^*, left) and *Pdf>mGluRA^RNAi^+Pdfr^RNAi^* (right) adult s-LN_v_s at CT9, 15, 21, and 3 on days 2–3 of DD immunostained for TIM and PDF. Examples for *Pdf>mGluRA^RNAi^+Pdfr^RNAi^* have been selected to show desynchronized LN_v_ clusters, but synchronized LN_v_s were also observed at each time point. (B) Histogram showing the percentage of synchronized (green) or desynchronized (red) s-LN_v_s in each cluster assayed by TIM staining in control (left) or *Pdf>mGluRA^RNAi^+Pdfr^RNAi^* (right) brains at CT 9, 15, and 21 on day 2 and CT3 on day 3 of DD. (C) Box plots showing quantification of desynchrony through measurement of ST DEV in TIM expression in adult s-LN_v_s in control and *Pdf>Pdfr^RNAi^*+*mGluRA^RNAi^* flies at CT3 and CT9 on day 3 in DD. *Pdf>Pdfr^RNAi^*+*mGluRA^RNAi^* significantly increased desynchrony measured by ST DEV in TIM or PDP1 expression at CT3 but not at CT9 compared to controls (ANOVA with Tukey's post hoc test, F_3,36_ = 5.313, *p* = 0.0039). (D) TIM expression in control (blue) or *Pdf>mGluRA^RNAi^+Pdfr^RNAi^* (red) s-LN_v_s. The amplitude of oscillation is dampened in *Pdf>mGluRA^RNAi^+Pdfr^RNAi^* compared to control LN_v_s (two-way ANOVA, genotype effect, F_1,82_ = 9.77, *p* = 0.0025). Error bars show SEM.

We next tested whether our findings from larvae held true for the more complicated adult circadian system. Because we observed the most dramatic effects on larval LN_v_ synchrony by simultaneously reducing *Pdfr* and *mGluRA* in LN_v_s, we measured the synchrony of s-LN_v_ molecular clocks in *Pdf>Pdfr^RNAi^*+*mGluRA^RNAi^* adult flies. We found that many more *Pdf>Pdfr^RNAi^*+*mGluRA^RNAi^* s-LN_v_ clusters were desynchronized than control s-LN_v_s (Figure 7A–C and Table S1), with extensive desynchrony detected at CT15 and CT21 on day 2 in DD and CT3 on day 3. TIM oscillations within *Pdf>Pdfr^RNAi^*+*mGluRA^RNAi^* s-LN_v_s also displayed a reduced amplitude compared to control s-LN_v_s, although the effect was less pronounced than in larvae (Figure 7D). We also observed significant desynchrony at CT3 when either *Pdfr* or *mGluRA* expression was reduced in LN_v_s (Figure S9A). We conclude that PDF and glutamate contribute to the robustness and synchrony of LN_v_ oscillations in adult flies as well as in larvae.

### Synchronizing Inputs to s-LN_v_s Regulate the Onset of Sleep

**Figure 8:**
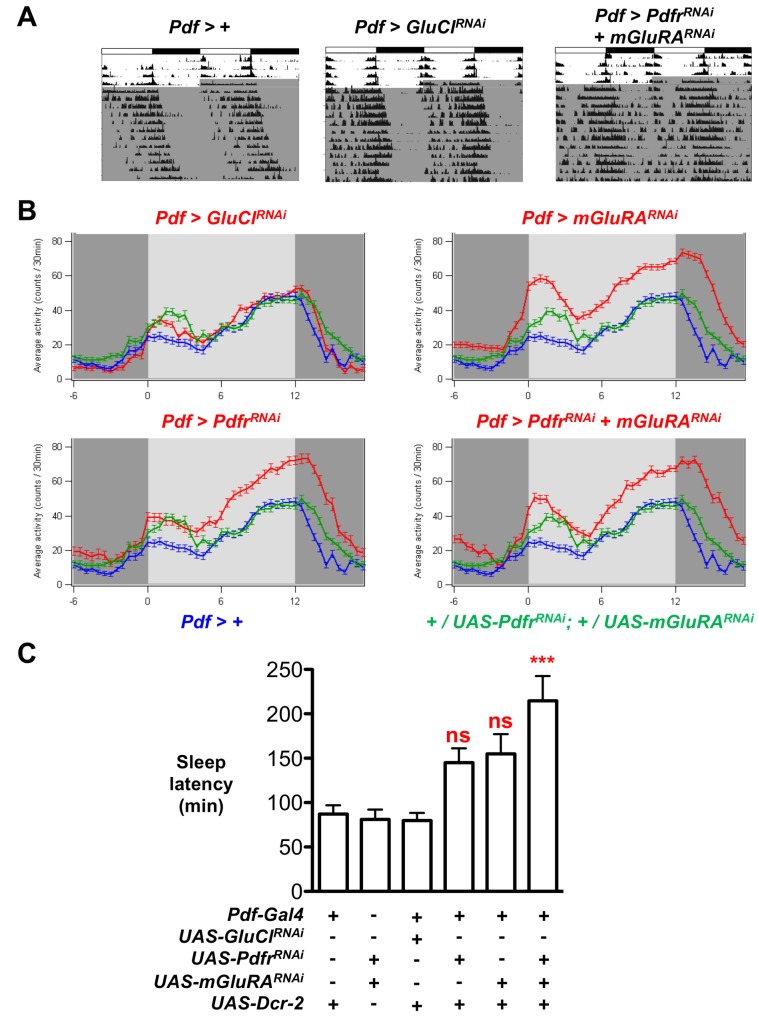
PdfR and mGluRA are required in LN_v_s for normal evening activity and timing of sleep onset. All experimental lines and *Pdf>+*control larvae also include *UAS-Dcr-2* for RNAi experiments, but this is omitted from written genotypes for simplicity. Error bars show SEM. *** *p*<0.001. (A) Locomotor activity was recorded for 3–4 days in LD cycles, followed by 10 days in DD (shaded area of actograms). Representative actograms are shown for *Pdf>+* control flies and for *Pdf>GluCl^RNAi^* and *Pdf>Pdfr^RNAi^+mGluRA^RNAi^* experimental flies. (B) Graphs show average locomotor activity over the first 5 days in DD. Each panel shows two control genotypes: *Pdf>+* (blue, *n* = 19) and *+/UAS-mGluRA^RNAI^*; +/*UAS-Pdfr^RNAi^* (green, *n* = 26). Experimental genotypes are shown in red. Top left: *Pdf>GluCl^RNAi^* (*n* = 37). Top right: *Pdf>mGluRA^RNAi^* (*n* = 54). Bottom left: *Pdf>Pdfr^RNAi^* (*n* = 33). Bottom right: *Pdf>Pdfr^RNAi^+mGluRA^RNAi^* (*n* = 37). Activity between ∼CT6 and 18 is elevated in *Pdf>mGluRA^RNAi^*, *Pdf>Pdfr^RNAi^*, and *Pdf>Pdfr^RNAi^+mGluRA^RNAi^* flies compared to controls or *Pdf>GluCl^RNAi^*. (C) Histogram shows the average sleep latency on the first day in DD. *Pdf>Pdfr^RNAi^+mGluRA^RNAi^* flies show significantly increased sleep latency compared to *Pdf>+*, *+/UAS-mGluRA^RNAI^*; +/*UAS-Pdfr^RNAi^*, and *Pdf>GluCl^RNAi^* controls (ANOVA F = 6.83, *p* = 0.0003).

We next tested whether *Pdf>Pdfr^RNAi^*+*mGluRA^RNAi^* flies displayed behavioral defects. We compared the locomotor activity of *Pdf>Pdfr^RNAi^*+*mGluRA^RNAi^* flies to parental flies and to *Pdf>GluCl* ^RNAi^ flies to control for nonspecific effects of RNAi in LN_v_s, as *GluCl* ^RNAi^ does not affect larval LN_v_ synchrony (Figure 3). Because *Pdf>Pdfr^RNAi^+mGluRA^RNAi^* flies have ∼24 h locomotor activity rhythms in DD, we conclude that s-LN_v_ desynchrony does not affect period length (Figure 8A and Table S2). However, we noticed that the activity of *Pdf>Pdfr^RNAi^+mGluRA^RNAi^* flies was much less consolidated than control or *Pdf>GluCl^RNAi^* flies, with bursts of activity visible in the subjective night when control flies are inactive (Figure 8A).

We calculated the average locomotor activity on the first 5 days in DD for each genotype. *Pdf>Pdfr^RNAi^*, *Pdf>mGluRA^RNAi^*, and *Pdf>Pdfr^RNAi^+mGluRA^RNAi^* flies displayed elevated levels of activity towards the end of subjective day and the beginning of subjective night (∼CT6–18) compared to control and *Pdf>GluCl^RNAi^* flies (Figure 8B). Thus altering PDF and/or glutamate inputs to LN_v_s increases nighttime activity.

To further quantify these differences in nighttime activity, we used standard measures of sleep. We found decreased overall sleep levels when *mGluRA* expression was reduced either alone or with *Pdfr* (Figure S9B). In contrast, reducing *Pdfr* expression alone had no significant effect on overall levels of sleep (Figure S9B). Thus, we conclude that glutamate signals to LN_v_s regulate sleep levels, whereas PDF signals between LN_v_s do not regulate sleep.

Next, we quantified the transition between wakefulness and sleep in the evening by measuring how quickly flies fell asleep after CT12 (sleep latency). To ensure that any effects on the timing of sleep onset did not result from subtle period length differences between genotypes (Table S2), we measured sleep latency only on day 1 in DD when the phase of locomotor activity between genotypes is minimally affected by small period differences. We found that *Pdf>Pdfr^RNAi^+mGluRA^RNAi^* flies showed a significant increase in sleep latency compared to all other genotypes (Figure 8C). Their average sleep latency of 213 min compared to 113 min for *UAS-Pdfr^RNAi^+UAS-mGluRA^RNAi^*/+ control flies exceeds the 30 min period length difference between these genotypes (Figure 8C and Table S2). We observed no significant effects when either *mGluRA* or *Pdfr* expression was reduced singly (Figure 8C).

Thus, we conclude that blocking PDF and glutamate inputs to LN_v_s increases evening activity and delays sleep onset timing. We did not observe a significant effect of reducing *mGluRA* or *Pdfr* expression on sleep latency under LD cycles (Figure S9C), consistent with LD cycles synchronizing larval LN_v_ clock oscillations (Figures 2D, 5D–E, S1C, and S3C). Although increased LN_v_ desynchrony may not cause the sleep latency defects observed, it is clear that normal *Pdfr* and *mGluRA* activity in LN_v_s is required for normal sleep in DD. However, it is possible that desynchrony and sleep latency defects are separate phenotypes resulting from abrogated intercellular communication between clock neurons.

## Discussion

### Synchronizing Larval Pacemaker Neurons Requires Two Signals

Feedback is an essential component in the molecular clocks that drive circadian behavior in animals [22]. Here we demonstrate the importance of feedback across the circadian neural network to synchronize individual clock neurons. We showed that larval LN_v_s require two signals that cooperate to synchronize their clocks: PDF released at dawn from LN_v_s themselves and glutamate released by DN_1_s at dusk. The PDF signal received by PdfR in DN_1_s presumably also sets the phase of the DN_1_ clock (Figure S2D) [8] to correctly time glutamate release that is then perceived by mGluRA in LN_v_s. Thus a feedback loop seems to exist at the circuit level, maintaining synchronized LN_v_ clocks in DD.

Our experiments also reveal that synchronization of larval pacemaker neurons is a very active process, as LN_v_ clocks were desynchronized 3 hours into the first subjective morning if they miss the dawn PDF signal. Consistent with the dual-synchronizer model, we see increased desynchrony when *mGluRA* and *Pdfr* expression is simultaneously reduced in LN_v_s (Figures 4B and S5A–C and Table S1).

### Dual Roles for Glutamate in the Circadian Circuit

A DN_1_ glutamate signal released around dusk is required for circadian rhythms of light avoidance when received by the ionotropic glutamate receptor GluCl in LN_v_s [9]. We now show that DN_1_ glutamate also promotes LN_v_ synchrony when received by the metabotropic glutamate receptor mGluRA in LN_v_s. Thus a single neurotransmitter plays two distinct roles in the *Drosophila* circadian circuit depending on the receptor that receives the signal in LN_v_s: a rapid behavioral response to light mediated via GluCl and longer-term regulation of the 24 hour molecular clock via mGluRA.

Although mGluRA is not required for light avoidance [9], we found that larvae with reduced expression of both *Pdfr* and *mGluRA* lose larval light avoidance rhythms. This is consistent with the loss of strong TIM protein oscillations in the LN_v_s of *Pdf>Pdfr^RNAi^+mGluRA^RNAi^* larvae. These defects in the LN_v_ molecular clock probably alter the timing of signals from LN_v_s and/or the phases of other clock neurons within the circuit. This contrasts with the role of GluCl, where glutamate received by GluCl directly regulates light avoidance by inhibiting the response of LN_v_s to ACh, independent of the LN_v_ molecular clock [9].

### Desynchronized Adult LN_v_s and Sleep

Synchronization of adult s-LN_v_s also depends on signaling via PdfR and mGluRA as >50% of s-LN_v_ clusters were desynchronized at three of the four timepoints measured when expression of both *Pdfr* and *mGluRA* was reduced in LN_v_s. However, TIM oscillations in adult s-LN_v_s were not as severely impaired as in larval LN_v_s. The increased complexity of the adult clock neural circuit probably adds signals from neurons not present in larvae that promote synchronized and robust clock protein oscillations in adult s-LN_v_s.

Because the molecular clock in adult *Pdf>mGluRA^RNAi^+Pdfr^RNAi^* flies still oscillates, it is not surprising that locomotor activity is also still largely rhythmic. However, the desynchrony and reduced amplitude of TIM oscillations in *Pdf>mGluRA^RNAi^+Pdfr^RNAi^* LN_v_s correlates with increased nighttime activity and sleep latency. Desynchrony and increased activity could be independent consequences of reduced glutamate and PDF receptivity in LN_v_s. An alternative possibility is that because the molecular clock regulates daily firing rhythms of clock neurons [34],, individual LN_v_s remain active at the wrong time of day in a desynchronized LN_v_ cluster, preventing sleep. Indeed, if LN_v_s are electrically coupled like SCN neurons [43], then firing of a single LN_v_ may cause the remaining LN_v_s in that cluster to fire earlier and/or later than programmed by their molecular clock. In addition, mistimed LN_v_ signals in *Pdf>Pdfr^RNAi^+mGluRA^RNAi^* flies will affect the phases of other clock neurons in the circuit, which could also disrupt sleep timing.

### Autonomy of the Molecular Clock

The loss of strong TIM protein oscillations in *Pdf>Pdfr^RNAi^+mGluRA^RNAi^* larval LN_v_s is surprising, as molecular clock oscillations in pacemaker neurons are often regarded as cell-autonomous. Our data extend conclusions from the SCN showing that the VIP receptor, VPAC2R, is required for synchronized molecular clocks [14]. By removing a second receptor simultaneously and by restricting our analyses to a defined subset of pacemaker neurons, we demonstrate that specifically blocking input signals to master pacemaker neurons has a dramatic effect on core clock protein oscillations.

We observed a much stronger effect on TIM than on PDP1 oscillations in *Pdf>mGluRA^RNAi^+Pdfr^RNAi^* larval LN_v_s. It may be that TIM oscillations are relatively easily modified, allowing information from outside the cell to be integrated into the molecular clock, whereas a more robust PDP1 oscillation prevents LN_v_s overreacting to external stimuli. It is well-documented that TIM can be regulated at the posttranslational level in addition to transcriptional control [22], whereas PDP1 protein levels closely follow *Pdp1* RNA levels [44]. We propose that external signals mediated via PdfR and mGluRA mainly regulate the clock posttranslationally, and this is supported by recent findings [45]–[47]. Testing this idea will require developing a combination of transcriptional and translational reporter genes.

### The Role of cAMP in Maintaining Clock Neuron Synchrony

VIP and VPAC2R synchronize the mammalian SCN in a cAMP/Ca^2+^-dependent manner [14],[16],[23]. VPAC2R is expressed more broadly than VIP, and some SCN neurons express both VIP and VPAC2R [48]. This is highly reminiscent of *Drosophila*, where *Pdfr* is found in both PDF+ and PDF– clock neurons [29]. Because VIP/VPAC2R and PDF/PdfR are functionally similar and because both mediate synchronization of pacemaker neurons, discoveries about the roles of PDF/PdfR in *Drosophila* should be relevant to understand how synchrony is maintained across circadian neural circuits in general. In both flies and mammals, a reciprocal relationship between synchrony and clock protein amplitude seems to allow pacemaker neurons to be more precise and robust timekeepers than individual neurons.

**Figure 9:**
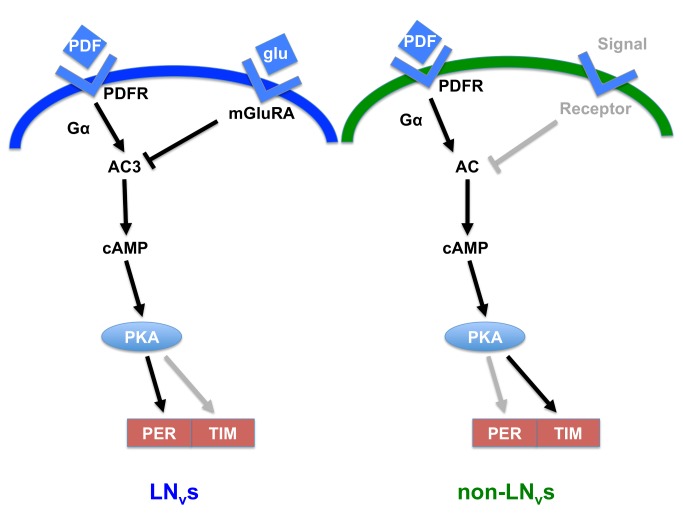
Model for regulation of cAMP levels and the molecular clock in clock neurons. Black arrows and text show established pathways; grey arrows and text reflect pathways inferred but not yet demonstrated. Left panel: In LN_v_s, PDF signals via PDFR and Gα/AC3 to boost intracellular cAMP [18]–[21]. In this study, we show that glutamate (glu) signals received via mGluRA reduce cAMP levels, likely by inhibiting AC3. Differentially timed release of PDF and glutamate signals results in cAMP rhythms. PKA responds to cAMP to increase stability of the PER/TIM dimer via PER [46] and likely also via TIM (data here and inferred from non-LN_v_s [45]). Right panel: In non-LN_v_ clock neurons, PDF signals via PDFR through Gα and unknown Adenyl cyclase(s) (AC) to boost intracellular cAMP. By analogy with what we show here for LN_v_s, we propose that an inhibitory signal released at a different time of day from PDF inhibits AC activity to generate a cAMP rhythm in non-LN_v_s. PKA responds to cAMP to increase stability of the PER/TIM dimer through TIM [45] and likely also PER (by analogy with LN_v_s [46]).

Our data reveal a remarkable degree of conservation of clock circuit properties between mammals and *Drosophila*, echoing the conserved molecular basis of the circadian clock. The mechanisms promoting LN_v_ synchrony in flies mirror the signaling pathways that make the SCN a more robust oscillator than other mammalian clock cells (reviewed in [4]). VIP and PDF are both required to synchronize the molecular clocks in different neurons, both promote robust oscillations of clock proteins within clock neurons, and they both likely signal through Gαs and Adenylate cyclase [4]. We have not yet determined the signaling pathways downstream of cAMP that link to clock protein oscillations, but they likely include PKA and/or Epac, which affect circadian rhythms in flies and mammals [23],[49],[50]. Recent data show that PKA lies downstream of PDF and cAMP in *Drosophila* clock neurons (see Figure 9). In addition, Epac can regulate MAP kinase signaling, which is interesting because MAP kinase has also been proposed to lie downstream of PDF [51].

Our data provide evidence that the external signals that drive cAMP oscillations are received at different times of day. In the SCN, the amplitude of the cAMP rhythm is amplified by increased VIP signaling at dawn. cAMP levels decrease at dusk via falling VIP release and a release of the inhibition of Gαi/o by RGS16 [5]. However, the behavioral phenotypes of *Rgs16^−/−^* mice are modest, suggesting that additional signaling mechanisms operate. Based on the similarity of the mammalian and *Drosophila* systems, we predict that a second signal released around dusk is also required for normal SCN function. Two possible signals are GABA [52] and glutamate perceived via its metabotropic receptor [53].

In *Drosophila*, different clock neuron groups respond to specific environmental inputs such as light or temperature [10],[11]. Thus the true function of the cAMP oscillator in flies and mammals may be to integrate information from diverse clock neurons into the molecular clocks of all clock neurons, generating a single time of day for an animal.

## Materials and Methods

### Fly Stocks

The following stocks used in this article have been described previously: *Pdf^01^* [15], *Pdfr^han5304^* [37], *cry13-Gal4* [54], *cry39-Gal4* [55], *Pdf-Gal80* [6], *tim(UAS)-Gal4* (referred to in the text as *tim-Gal4* for simplicity [56]), *Pdfr-Gal4* ^GMR18F07^ [57], *UAS-Dti* [58], *Pdf_0.5_-Gal4* [59], *UAS-CD8::GFP* [60], *UAS-Epac1-camps* [19], *UAS-mGluRA^RNAi^* [32], *UAS-Dicer-2* [61], *UAS-Gad1* [33], *UAS-GluCl^RNAi^* (v105724) [9],[62], *UAS-Pdfr^RNAi^* (v42724) [61], *UAS-Shi^ts^* [36], *mGluRA^112b^* [63], *UAS-AC3^Vienna RNAi^* (v33217), and *UAS-AC3^TRiP RNAi^* (JF03041) [21]. *UAS-mGluRA^RNAi^*, *UAS-Pdfr^RNAi^*, *Pdfr^han^*, *mGluRA^112b^*, *Pdf^01^*, *[Pdf-Gal4; Dcr-2]*, *[Pdf-Gal80; cry-Gal4]*, *UAS-Dti*, and *UAS-Shi^ts^* stocks all carry the *ls-tim* allele [64]; thus, differences in TIM expression observed are not due to an inability of flies to express specific *tim* isoforms.

### Immunocytochemistry

All immunocytochemistry was carried out as in [44]. We used the following antibodies: rat αTIM (from Amita Sehgal), rabbit αPDP1 [44], mouse αPDF [65], and rabbit αGFP (Sigma, St. Louis, MO). Images were scanned on a Leica SP2, SP6, or SP8 confocal microscope, with the same microscope used for a single experiment. The beginning and end of TIM staining was used to establish the limits of confocal stacks. The mean staining intensity for each channel for each neuron in every confocal stack was quantified using FIJI (http://pacific.mpi-cbg.de/wiki/index.php/Main_Page), with background levels of staining for each channel subtracted to control for variation in staining between brains. For each time course, the mean staining intensities for all LN_v_s in each brain lobe were averaged to give a single value for an LN_v_ cluster. The average staining intensities per brain were then averaged to generate the time courses shown.

We used two methods to measure LN_v_ synchrony. In a simple binary method, we used a cutoff of 20 arbitrary units (au) above background to determine if a cell produced TIM or PDP1 or not. We chose 20 au as it is the lowest number where protein levels are convincingly visible above background. An LN_v_ cluster was then scored as “desynchronized” if they contained a mixture of LN_v_s with and without detectable TIM or PDP1, or “synchronized” if all four LN_v_s were the same. To more precisely quantify desynchrony, we also calculated the standard deviation in TIM or PDP1 mean staining intensities between individual LN_v_s within a single LN_v_ cluster, producing a standard deviation in TIM or PDP1 staining intensity to use as a proxy for the level of desynchrony, allowing statistical comparisons of the data.

### Behavioral Assays

Larval light avoidance assays were carried out as in [9]. For adult locomotor activity experiments, adults were entrained to 12∶12 LD cycles at 25°C for at least 3 days before transfer to DD. Locomotor activity was recorded using the DAM system (TriKinetics, Waltham, MA).

### cAMP Measurements

Basal levels of Epac1-camps FRET were used to measure cAMP levels. Larval brains were dissected and mounted in hemolymph-like saline. To minimize the time from dissection to imaging (∼1 hour), different genotypes were removed from DD, dissected, and scanned in the same order. LN_v_ projections were imaged for CFP (460–490 nm) and YFP (528–603 nm) on an SP5 Leica confocal using a TD 458/514/594 dichroic 63× lens and 3× digital zoom at 100 Hz and 1024×1024 resolution, after excitation with a 458 nm laser. CFP and YFP levels were quantified using the Leica software. Background measurements of CFP and YFP were subtracted from raw CFP and YFP measurements and an average CFP to YFP ratio calculated for each image. For LN_v_ projections, five boutons in each image were quantified for CFP and YFP as above and averaged to give a value per projection.

Live cAMP imaging was performed on larval LN_v_s as described in [66]. Briefly, larval brains were dissected in hemolymph-like saline and mounted to the bottom of a 35-mm Falcon culture dish lid (Becton Dickenson Labware, Franklin Lakes, NJ), fitted with a Petri Dish Insert (PDI, Bioscience Tools, San Diego, CA). Brains were allowed to settle for 5–10 min to reduce movement during imaging. Images were acquired on an Olympus FV1000 laser-scanning microscope (Olympus, Center Valley, PA) through a 60× (1.1N/A W, FUMFL N) Objective (Olympus, Center Valley, PA) using Fluoview software (Olympus). The Epac1-camps FRET sensor was imaged by scanning frames at 1 Hz with a 440-nm laser. An SDM510 dichroic mirror was used to separate CFP and YFP emission. Regions of interest were drawn around single LN_v_ cell bodies in Fluoview. Peptides were bath applied using a micropipette after 30 s of baseline imaging. PDF was dissolved in 0.01% DMSO and vehicle controls consisted of 0.01% DMSO delivered at the same volume as peptide applications (45 µL bath application into 405 µL hemolymph-like saline). The lowest PDF dose that evoked a consistent response (100 nM) was used to assay differential responses of LN_v_s in which PDF or glutamate receptors had been knocked down in Figure 6. PDF was used at 100 µM in Figure S2. For each assay, no less than five larvae were imaged. Only one hemisphere was imaged per brain, and 1–4 LN_v_ were imaged per brain. Processing and analysis of Epac1-camps data was as described [67].

### Sleep Analysis

Fly locomotor activity was recorded in 5 min bins, using the DAM system (TriKinetics). Data analysis was performed using custom-written scripts in IgorPro (Wavemetrix). Sleep was defined as periods of immobility >5 min. Sleep latency was calculated for each fly on each day as the time from CT12 until the first sleep bout. Locomotor activity was calculated as the average number of beam crossings per 30 min bins and averaged for each genotype over the first 5 days in DD.

## Supporting Information

Figure S1 **PDF signaling is required for LN_v_ synchronization in DD.** (A) Histograms showing the number of LN_v_s expressing TIM in each brain lobe in control, *Pdf^01^*, and *Pdfr^han^* larvae at CT3 and CT9. Because no TIM+ LN_v_s were detected in control brains at either time point, all LN_v_ clusters were synchronized (green). TIM was detected in one, two, or three LN_v_s at CT3 in 50% of *Pdf^01^* mutant brains and in 58% of *Pdfr^han^* mutant brains; these are defined as desynchronized (red). No *Pdf^01^* or *Pdfr^han^* LN_v_s expressed TIM at CT9; thus all LN_v_ clusters were synchronized. (B) Histograms showing the number of LN_v_s expressing PDP1 in each brain lobe in control and *Pdfr^han^* larvae at CT3 and CT9. No PDP1+ LN_v_s were detected in control brains at either time point; thus, all LN_v_ clusters were synchronized (green). In *Pdfr^han^* larvae, PDP1 was detected in one, two, or three LN_v_s in 50% of brains examined at CT3; these are desychronized. No *Pdfr^han^* LN_v_s expressed PDP1 at CT9, and thus, all LN_v_ clusters were synchronized. (C) Histograms showing the number of LN_v_s expressing TIM in each brain lobe in control, *Pdfr^han^*, and *Pdf^01^* larvae at ZT3.
(TIF)

Figure S2 **Responses to PDF in DN1s and LNvs.** Error bars represent SEM. * *p*<0.05; ** *p*<0.01; *** *p*<0.005. (A) Left: Average traces showing the responses of *cry+ 39>Epac1-camps* larval DN_1_s to 10 µM PDF (green), 10 µM PDF+2 µM TTX (purple), vehicle (blue), or vehicle+2 µM TTX (black). Shaded area around each line shows SEM. Brains were incubated in TTX for 20 min prior to the PDF application. Right: Histogram shows the maximum percentage change of CFP/YFP after bath application of Vehicle (Veh) or 10 µM PDF peptide ±2 µM TTX. DN_1_s respond to PDF more strongly than to vehicle both without TTX (*p* = 0.0033) or with TTX (*p* = 0.0055). The *p* values were calculated using a multiple *t* test with Tukey's analysis. (B) *Pdfr-Gal4* ^GMR18F07^ was used to express UAS-GFP (green). Larvae were dissected at ZT21 and stained with PDF (blue) and PDP1 (red). This *Pdfr* enhancer-Gal4 localizes to DN1s but not DN_2_s. (C) Left: Average traces showing the responses of control (*Pdf>Epac1-camps*, blue), RNAi control (*Pdf>babo^RNAi^; UAS-Epac1-camps*, green), or *Pdfr^RNAi^* (*Pdf>Pdfr^RNAi^*+*Epac1-camps*, red) LN_v_s to application of 10 µM PDF. Average response of LN_v_s to application of vehicle is shown in black. Shaded area around each line shows SEM. Right: Histogram shows the maximum percentage change of CFP/YFP after bath application of 10 µM PDF peptide. Expression of *Pdfr^RNAi^* (*Pdf>Pdfr^RNAi^*+*Epac1-camps*) significantly reduces the maximum percentage change of CFP/YFP upon PDF application compared to control LN_v_s. Controls were sensor only (*Pdf>UAS-Epac1-camps*; *p* = 0.0045) and a line expressing a control RNAi (*Pdf>babo^RNAi^ UAS-Epac1-camps*; *p* = 0.0426). The *p* values were calculated using the Mann–Whitney nonparametric *t* test. (D) DN_1_ TIM oscillations on days 2 and 3 in DD show an altered phase in *Pdfr^han^* mutants compared to controls (two-way ANOVA, significant interaction between genotype and time, F_3,192_ = 3.2, *p* = 0.03).
(TIF)

Figure S3 **LN_v_ and non-LN_v_ clock neurons maintain LN_v_ synchrony.** For all RNAi experiments, *Gal4/+* control and experimental lines include *UAS-Dcr-2*. Error bars represent SEM. * *p*<0.05; ** *p*<0.01; *** *p*<0.001; **** *p*<0.0001. (A) Histograms showing the percentage of LN_v_ clusters synchronized (green) or desynchronized (red) for TIM or PDP1 expression at CT3 in (left top panel) *Pdf>+; +/UAS-Pdfr^RNAi^*; *Pdf>Pdfr^RNAi^*, (left bottom panel) *tim >+; UAS-Pdfr^RNAI^/Pdf-Gal80; tim-Gal4*>*Pdfr^RNAi^*, *Pdf-Gal80*, and (right) *DN_1_>+; UAS-Dti*/+; and DN_1_ ablated larvae (*DN_1_>Dti*). (B and C) Box plots showing the distribution of ST DEV in PDP1 expression, with whiskers representing 95% confidence interval. (B) *Pdf>Pdfr^RNAi^* significantly increase ST DEV in PDP1 levels within an LN_v_ cluster compared to both parental controls (ANOVA F_2,49_ = 7.809, *p* = 0.0011), reflecting increased desynchrony. By ANOVA with Tukey's post hoc test, *tim-Gal4; Pdf-Gal80*>*Pdfr^RNAi^* is significantly different only from *tim>+*control LN_v_s (F_2,51_ = 4.434, *p* = 0.017). However, by Student's *t* test, levels of PDP1 are also significantly increased in *tim-Gal4; Pdf-Gal80*>*Pdfr^RNA^* compared to *UAS-Pdfr^RNAi^/Pdf-Gal80* controls (*p* = 0.046). (C) ST DEV in PDP1 levels between LN_v_s in each brain lobe at ZT3 and ZT9 in LD and CT3 and CT9 on day 3 in DD. Statistical comparisons by ANOVA with Tukey's post hoc test show a significant increase in ST DEV in PDP1 expression in *DN_1_>Dti* larvae compared to controls at CT3 only (F_2,49_ = 8.59, *p* = 0.0006). (D) TIM and (E) PDP1 immunostaining was quantified for LN_v_s of Control (*+/UAS-Dti*; blue) and DN_1_-ablated (*DN_1_>Dti*, red) larval brains in ZT and days 2 and 3 in DD. DN_1_s are not required for LN_v_s to oscillate in DD (TIM, ANOVA, F_3,42_ = 12.66, *p*<0.0001, and PDP1, ANOVA, F_3,28_ = 23.71, *p*<0.0001). However, TIM levels were significantly higher at CT3 on days 2 and 3 in *DN_1_>Dti* larvae compared to controls (Student's *t* test, *p* = 0.0004 and *p*<0.0001, respectively), and PDP1 levels were significantly higher at CT3 on day 3 in *DN_1_>Dti* larvae compared to controls (Student's *t* test, *p* = 0.022).
(TIF)

Figure S4 **Desynchronization of larval LN_v_s with altered glutamate signaling.** For RNAi experiments, all experimental lines and *Pdf>+*control lines (*Pdf>+*) include *UAS-Dcr-2*. Whiskers represent 95% confidence interval. Error bars represent SEM. * *p*<0.05; ** *p*<0.01; *** *p*<0.001. (A) TIM levels in LN_v_s were compared between control (*UAS-Gad1*, blue) and *DN_1_>Gad1* (green) larval brains. Reducing DN_1_ glutamate signaling through *Gad1* misexpression (*DN_1_>Gad1*) leaves TIM oscillations intact in LN*_v_*s (ANOVA, F_3,52_ = 19.63, *p*<0.0001) but increases TIM levels at CT3 (Student's *t* test, *p*<0.0001). (B) TIM levels in LN_v_s were compared between control (*+/UAS-GluCl^RNAi^*, blue) and *Pdf>GluCl^RNAi^* (red) larvae. Reducing *GluCl* levels in LN_v_s had no effect on TIM oscillations (ANOVA F_3,43_ = 78.99, *p*<0.0001) or TIM expression at CT3 (Student's *t* test, *p* = 0.34). (C) Box plots showing quantification of desynchrony through measurement of ST DEV in PDP1 expression in larval LN_v_s in control, *DN_1_>Gad1*, *Pdf>GluCl^RNAi^*, and *Pdf>mGluRA^RNAi^* larvae at CT3 on day 3 in DD. *DN_1_>Gad1* (Student's *t* test, *p* = 0.0035) and *Pdf>mGluRA^RNAi^* (ANOVA with Tukey's post hoc test, F_2,50_ = 10.54, *p* = 0.0002) significantly increase the ST DEV in PDP1 levels, and therefore desynchrony, compared to parental controls, whereas *Pdf>GluCl^RNAi^* does not (ANOVA with Tukey's post hoc test, F_2,39_ = 0.11, *p* = 0.90). (D) Box plots showing quantification of desynchrony through measurement of ST DEV in TIM (left) and PDP1 (right) in *mGluRA^112b^* mutants and controls. The ST DEV of TIM (Student's *t* test, *p* = 0.0022) and PDP1 (Student's *t* test, *p* = 0.013) is significantly increased in *mGluRA^112b^* mutants compared to controls (*mGluRA112b/+*).
(TIF)

Figure S5 **Signaling via mGluRA and PdfR synchronizes LN_v_ clocks.** For RNAi experiments, all experimental lines and *Pdf>+* control lines include *UAS-Dcr-2*. Error bars represent SEM. **** *p*<0.0001. (A) Histogram showing the number of synchronized (green) or desynchronized (red) LN_v_ clusters in control (*+/UAS-mGluRA^RNAI^*; +/*UAS-Pdfr^RNAi^*) or *Pdf>mGluRA^RNAi^+Pdfr^RNAi^* larval brains, determined by PDP1 staining at CT3. (B and C) Box plots quantifying desynchrony by measuring ST DEV in TIM (B) and PDP1 (C) expression in larval LN_v_s in control (*+/UAS-mGluRA^RNAI^*; +/*UAS-Pdfr^RNAi^*) and *Pdf>Pdfr^RNAi^*+*mGluRA^RNAi^* larvae at CT3 and CT9 on day 3 in DD. Whiskers represent 95% confidence interval. *Pdf>Pdfr^RNAi^*+*mGluRA^RNAi^* significantly increased desynchrony as measured by ST DEV in TIM or PDP1 expression at CT3 but not CT9 compared to controls (ANOVA with Tukey's post hoc test; TIM, F_3,47_ = 31.96, *p*<0.0001, and PDP1, F_3,47_ = 23.43, *p*<0.0001). (D) Average PDP1 levels of control (blue) and *Pdf>mGluRA^RNAi^+Pdfr^RNAi^* (green) LN_v_s. PDP1 oscillates relatively normally in *Pdf>mGluRA^RNAi^+Pdfr^RNAi^* larval LN_v_s (two-way ANOVA, no significant genotype effect, F_1,82_ = 0.15, *p* = 0.6970). Average TIM (E) and PDP1 (F) levels are shown for *Pdf>Pdfr^RNAi^* (red) and *Pdf>mGluRA^RNAi^* (green) LN_v_s in DD on days 2 and 3. *Pdf>mGluRA^RNAi^* and *Pdf>Pdfr^RNAi^* larval LN_v_s display similar TIM and PDP1 oscillations. TIM, two-way ANOVA, no significant genotype effect (F_1,80_ = 0.24, *p* = 0.6224) but a significant time effect (F_3,80_ = 19.98, *p*<0.0001). For PDP1, no significant genotype effect (F_1,79_ = 1.15, *p* = 0.2876) but a significant time effect (F_3,79_ = 13.87, *p*<0.0001).
(TIF)

Figure S6 **Dawn PDF and Dusk glutamate signals alter LN_v_ PDP1 expression.** All statistical comparisons are by ANOVA with Tukey's post hoc test unless otherwise stated. Error bars represent SEM. Whiskers represent 95% confidence interval. * *p*<0.05; ** *p*<0.01; *** *p*<0.005. (A) Histograms showing the percentage of LN_v_ clusters showing synchronized/desynchronized PDP1 expression in control or *DN_1_>shi^ts^* LN_v_s after a 6 hour 31°C heat pulse centered at CT12 or CT24. (B) Box plots representing the ST DEV of PDP1 expression in LN_v_s of control or *DN_1_>shi^ts^* larvae dissected at CT3 on day 3 of DD after a 31°C heat pulse centered at CT12 or CT24 on day 2 of DD. A heat pulse at CT12 significantly increased the ST DEV in PDP1 expression of *DN_1_>Shi^ts^* larval LN_v_ clusters (Student's *t* test, CT12 versus 24, *p*<0.01), but did not affect controls. (C) Larval LN_v_s were immunostained for PDP1 at ZT3 and at CT3 on days 1 and 2 of DD in Control (*+/UAS-Dti*), *DN_1_>Dti*, and *Pdf^01^* mutants. DN_1_ ablation or the *Pdf^01^* mutation do not affect LN_v_ PDP1 levels at ZT 3 (F_2,34_ = 1.70, *p* = 0.2). *Pdf^01^* increases TIM expression in LN_v_s on the first day of DD, whereas *DN_1_>Dti* does not (F_2,38_ = 8.62, *p* = 0.0008). (D) Desynchrony of LN_v_s in ZT and on the first and second days of DD was quantified by measuring ST DEV of PDP1 expression in Con (*+/UAS-Dti*), *DN_1_>Dti*, and *Pdf^01^* mutants. There is no difference between genotypes at ZT3 (F_2,34_ = 2.89, *p* = 0.07). ST DEV in PDP1 is significantly higher in *Pdf^01^* LN_v_s compared to control or *DN_1_>Dti* LN_v_s on the first day of DD, reflecting increased desynchrony (F_2,38_ = 4.62, *p* = 0.016). *DN_1_>Dti* increases desynchrony as measured by PDP1 ST DEV only on day 2 in DD (Student's *t* test, *p* = 0.041).
(TIF)

Figure S7 **Dose response of larval LN_v_s to bath-applied PDF.** Error bars show SEM. * *p*<0.05; ** *p*<0.01; *** *p*<0.001. (A) Averaged Epac-1-camps CFP/YFP ratio responses to bath application (triangle) of a range of PDF concentrations and vehicle. Sample sizes were as follows: vehicle, seven LN_v_ cell bodies imaged from five brains (7, 5), PDF 10^−8^ M: (12, 5), PDF 10^−7^ M: (15, 6), PDF 3×10^−7^ M: (17, 6), PDF 10^−6^ M: (13, 5), PDF 3×10^−6^ M: (14, 5), and PDF 10^−5^ M: (16, 6). Error bars represent SEM. (B) Comparison of mean maximum Epac-1-camps CFP/YFP ratio changes between 0 and 240 s (dashed line in A) for the neurons shown in (A). cAMP responses to the various PDF doses were compared by means of a Kruskal–Wallis one-way ANOVA, and a Dunn's multiple comparison test was performed to determine which treatments within the group of compounds tested produced responses significantly different from vehicle controls. (C) Data from (B) fitted as a dose–response curve. The EC_50_ is 1.1×10^−7^ M PDF.
(TIF)

Figure S8 **Adenylate cyclase 3 is required in LN_v_s for synchrony.** Desynchrony data are calculated from 3–5 independent experiments, each consisting of at least four brains. Total number of LN_v_ clusters analyzed are in Table S1. Error bars show SEM. Whiskers represent 95% confidence. * *p*<0.05; ** *p*<0.01. (A) Representative images of LN_v_s in control larvae (*UAS-RNAi transgene*/+) or in larvae with LN_v_s expressing one of two independent RNAi transgenes targeting AC3 (*Pdf>AC3^TRiP^* or *Pdf>AC3^Vienna^*) immunostained for PDF (green), TIM (red), and PDP1 (blue) at CT3 on day 3 in DD. The lower panels for each genotype are the same images with the green channel (PDF) removed and replaced by a dashed white line outlining LN_v_s. (B) Histograms show the percentage of LN_v_ clusters in which TIM (left) or PDP1 (right) was detected in either none or all four of the four LN_v_s (“synchronized,” green bars) or in one, two, or three LN_v_s (“desynchronized,” red bars). Box plots showing the ST DEV in (C) TIM or (D) PDP1 expression as in Figure 1. Statistical comparisons show reducing AC3 expression in LN_v_s via *Pdf>AC3^TRiP^* or *Pdf>AC3^Vienna^* significantly increases the ST DEV of TIM and PDP1 levels compared to respective controls, reflecting increased desynchrony.
(TIF)

Figure S9 **Effects of reduced** ***PdfR*** **and** ***mGluRA*** **signaling in adult LN_v_s.** Error bars represent SEM. Statistics shown represent the least significant difference to all control genotypes as calculated by ANOVA with Tukey's post hoc test. *** *p*<0.001. (A) Histogram showing the percentage of s-LN_v_ clusters showing synchronized (green) or desynchronized (red) TIM expression in Control (*Pdf>+*) flies or flies expressing RNAi transgenes targeting *mGluRA*, *Pdfr*, or *GluCl*. (B) Histogram shows the percentage of time spent asleep over the first 5 d in DD. *Pdf>mGluRA^RNAi^* (ANOVA F = 31.32, *p*<0.0001) and *Pdf>Pdfr^RNAi^+mGluRA^RNAi^* (ANOVA F = 23.84, *p*<0.0001) flies show significantly reduced time spent asleep compared to *Pdf>+*, *+/UAS-mGluRA^RNAI^*; +/*UAS-Pdfr^RNAi^*, or *Pdf>GluCl^RNAi^* flies. (C) Histogram shows the average sleep latency in LD. By ANOVA, there are no significant differences in sleep latency between *Pdf>mGluRA^RNAi^*, *Pdf>Pdfr^RNAi^*, or *Pdf>Pdfr^RNAi^+mGluRA^RNAi^* flies and *UAS-Pdfr^RNAi^+UAS-mGluRA^RNAi^/+* controls.
(TIF)

Table S1 **Number of LN_v_s expressing TIM or PDP1 in each LN_v_ cluster analyzed.** Numbers indicate the number of clusters with zero, one, two, three, or four LN_v_s expressing TIM or PDP1 for each genotype.
(PDF)

Table S2 **Behavioral periods and strengths of behavioral rhythms in adult flies with altered glutamate and PDF receptor expression in LN_v_s.**
(PDF)

## Abbreviations

cAMP: cyclic AMP
CLK: Clock
CT: Circadian time
CYC: Cycle
DD: constant darkness
*DN_1_>X*: cry-Gal4, Pdf-Gal80 *expressing UAS transgene X*
DN_1_s: dorsal neurons group 1
Gad1: Glutamate decarboxylase 1
GluCl: Glutamate-gated chloride channel
GPCR: G-protein coupled receptor
LD: light–dark cycles
LN_v_s: ventral lateral neurons
mGluRA: metabotropic Glutamate Receptor
*Pdf>X*: *Pdf-Gal4* expressing UAS transgene X
PDF: Pigment Dispersing Factor
PdfR: PDF Receptor
PDP1: PAR-domain protein 1
PER: period
SCN: suprachiasmatic nucleus
*tim>X*: *tim-Gal4* expressing UAS transgene X
TIM: timeless
VIP: vasoactive intestinal peptide
VPAC: VIP receptor
ZT: Zeitgeber time
