# Supplementary figures and images for: Differentially Timed Extracellular Signals Synchronize Pacemaker Neuron Clocks
Source: PLoS Biol. 2014 Sep 30;12(9):e1001959. doi: 10.1371/journal.pbio.1001959 (PMC4181961; doi:10.1371/journal.pbio.1001959)

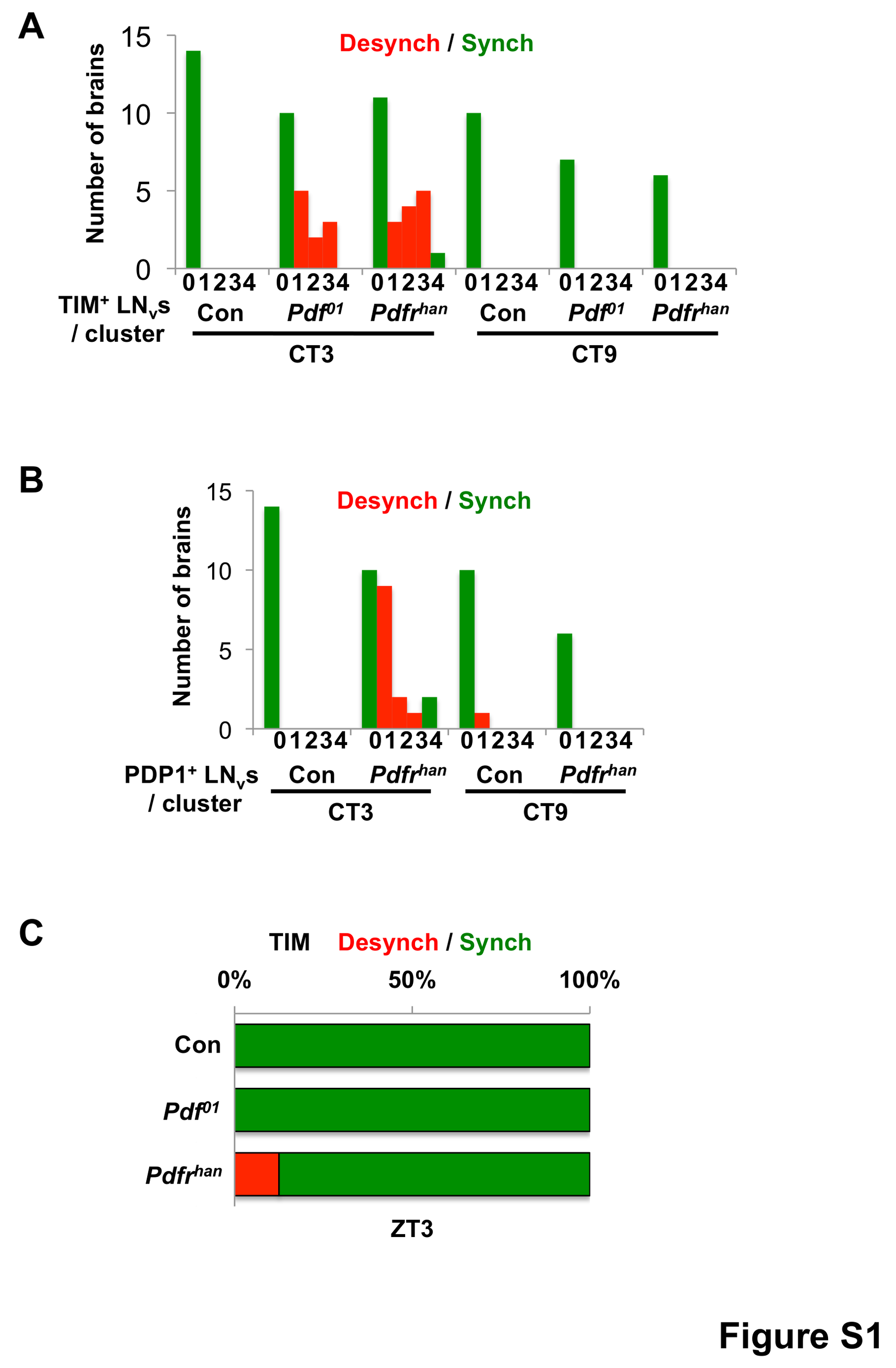

Supplement: Figure S1 — PDF signaling is required for LNv synchronization in DD. (A) Histograms showing the number of LNvs expressing TIM in each brain lobe in control, Pdf01, and Pdfrhan larvae at CT3 and CT9. Because no TIM+ LNvs were detected in control brains at either time point, all LNv clusters were synchronized (green). TIM was detected in one, two, or three LNvs at CT3 in 50% of Pdf01 mutant brains and in 58% of Pdfrhan mutant brains; these are defined as desynchronized (red). No Pdf01 or Pdfrhan LNvs expressed TIM at CT9; thus all LNv clusters were synchronized. (B) Histograms showing the number of LNvs expressing PDP1 in each brain lobe in control and Pdfrhan larvae at CT3 and CT9. No PDP1+ LNvs were detected in control brains at either time point; thus, all LNv clusters were synchronized (green). In Pdfrhan larvae, PDP1 was detected in one, two, or three LNvs in 50% of brains examined at CT3; these are desychronized. No Pdfrhan LNvs expressed PDP1 at CT9, and thus, all LNv clusters were synchronized. (C) Histograms showing the number of LNvs expressing TIM in each brain lobe in control, Pdfrhan, and Pdf01 larvae at ZT3. (TIF) [file pbio.1001959.s001.tif]

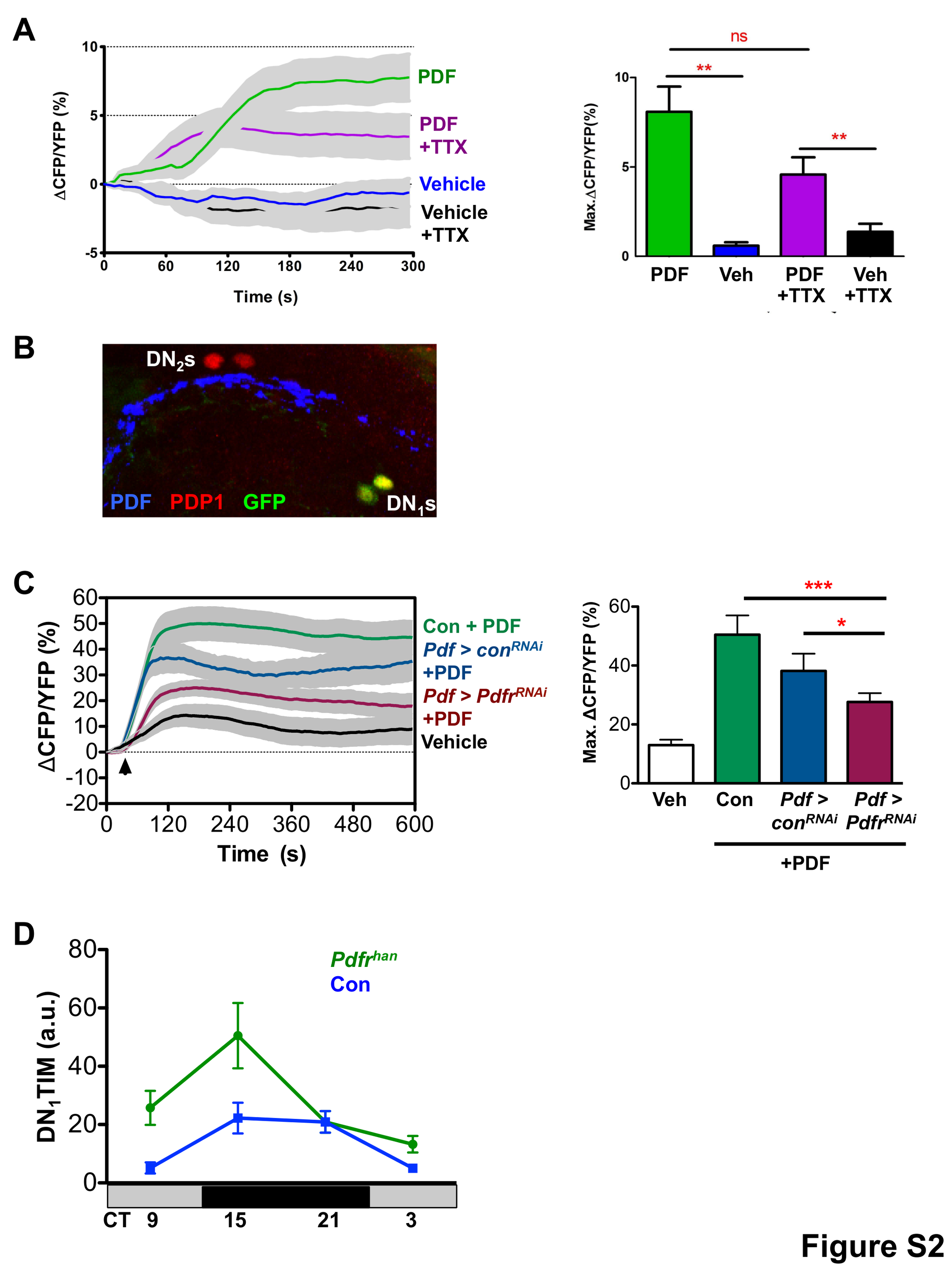

Supplement: Figure S2 — Responses to PDF in DN1s and LNvs. Error bars represent SEM. * p<0.05; ** p<0.01; *** p<0.005. (A) Left: Average traces showing the responses of cry+ 39>Epac1-camps larval DN1s to 10 µM PDF (green), 10 µM PDF+2 µM TTX (purple), vehicle (blue), or vehicle+2 µM TTX (black). Shaded area around each line shows SEM. Brains were incubated in TTX for 20 min prior to the PDF application. Right: Histogram shows the maximum percentage change of CFP/YFP after bath application of Vehicle (Veh) or 10 µM PDF peptide ±2 µM TTX. DN1s respond to PDF more strongly than to vehicle both without TTX (p = 0.0033) or with TTX (p = 0.0055). The p values were calculated using a multiple t test with Tukey's analysis. (B) Pdfr-Gal4 GMR18F07 was used to express UAS-GFP (green). Larvae were dissected at ZT21 and stained with PDF (blue) and PDP1 (red). This Pdfr enhancer-Gal4 localizes to DN1s but not DN2s. (C) Left: Average traces showing the responses of control (Pdf>Epac1-camps, blue), RNAi control (Pdf>baboRNAi; UAS-Epac1-camps, green), or PdfrRNAi (Pdf>PdfrRNAi+Epac1-camps, red) LNvs to application of 10 µM PDF. Average response of LNvs to application of vehicle is shown in black. Shaded area around each line shows SEM. Right: Histogram shows the maximum percentage change of CFP/YFP after bath application of 10 µM PDF peptide. Expression of PdfrRNAi (Pdf>PdfrRNAi+Epac1-camps) significantly reduces the maximum percentage change of CFP/YFP upon PDF application compared to control LNvs. Controls were sensor only (Pdf>UAS-Epac1-camps; p = 0.0045) and a line expressing a control RNAi (Pdf>baboRNAi UAS-Epac1-camps; p = 0.0426). The p values were calculated using the Mann–Whitney nonparametric t test. (D) DN1 TIM oscillations on days 2 and 3 in DD show an altered phase in Pdfrhan mutants compared to controls (two-way ANOVA, significant interaction between genotype and time, F3,192 = 3.2, p = 0.03). (TIF) [file pbio.1001959.s002.tif]

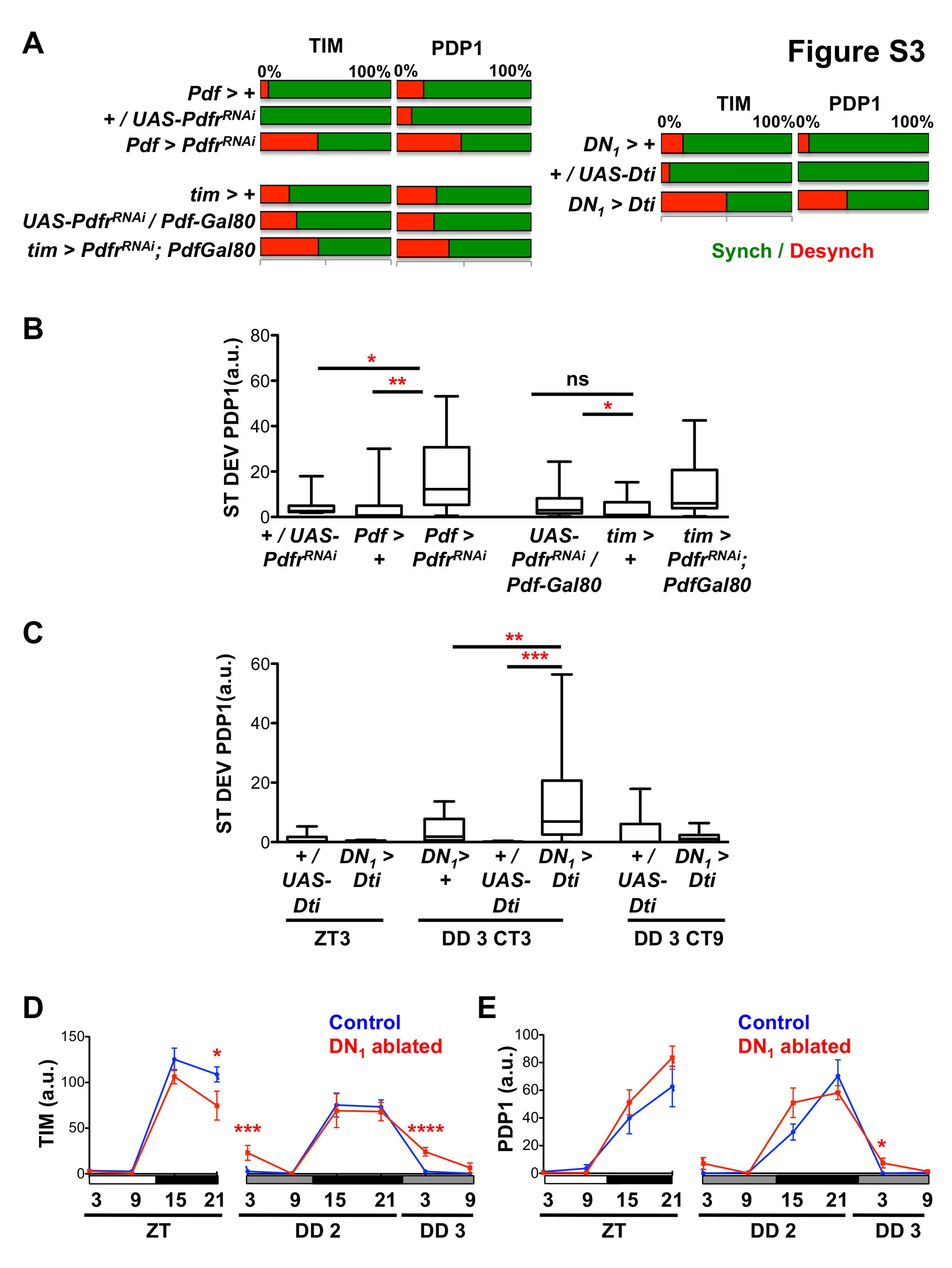

Supplement: Figure S3 — LNv and non-LNv clock neurons maintain LNv synchrony. For all RNAi experiments, Gal4/+ control and experimental lines include UAS-Dcr-2. Error bars represent SEM. * p<0.05; ** p<0.01; *** p<0.001; **** p<0.0001. (A) Histograms showing the percentage of LNv clusters synchronized (green) or desynchronized (red) for TIM or PDP1 expression at CT3 in (left top panel) Pdf>+; +/UAS-PdfrRNAi; Pdf>PdfrRNAi, (left bottom panel) tim >+; UAS-PdfrRNAI/Pdf-Gal80; tim-Gal4>PdfrRNAi, Pdf-Gal80, and (right) DN1>+; UAS-Dti/+; and DN1 ablated larvae (DN1>Dti). (B and C) Box plots showing the distribution of ST DEV in PDP1 expression, with whiskers representing 95% confidence interval. (B) Pdf>PdfrRNAi significantly increase ST DEV in PDP1 levels within an LNv cluster compared to both parental controls (ANOVA F2,49 = 7.809, p = 0.0011), reflecting increased desynchrony. By ANOVA with Tukey's post hoc test, tim-Gal4; Pdf-Gal80>PdfrRNAi is significantly different only from tim>+control LNvs (F2,51 = 4.434, p = 0.017). However, by Student's t test, levels of PDP1 are also significantly increased in tim-Gal4; Pdf-Gal80>PdfrRNA compared to UAS-PdfrRNAi/Pdf-Gal80 controls (p = 0.046). (C) ST DEV in PDP1 levels between LNvs in each brain lobe at ZT3 and ZT9 in LD and CT3 and CT9 on day 3 in DD. Statistical comparisons by ANOVA with Tukey's post hoc test show a significant increase in ST DEV in PDP1 expression in DN1>Dti larvae compared to controls at CT3 only (F2,49 = 8.59, p = 0.0006). (D) TIM and (E) PDP1 immunostaining was quantified for LNvs of Control (+/UAS-Dti; blue) and DN1-ablated (DN1>Dti, red) larval brains in ZT and days 2 and 3 in DD. DN1s are not required for LNvs to oscillate in DD (TIM, ANOVA, F3,42 = 12.66, p<0.0001, and PDP1, ANOVA, F3,28 = 23.71, p<0.0001). However, TIM levels were significantly higher at CT3 on days 2 and 3 in DN1>Dti larvae compared to controls (Student's t test, p = 0.0004 and p<0.0001, respectively), and PDP1 levels were significantly higher at CT3 on d [file pbio.1001959.s003.tif]

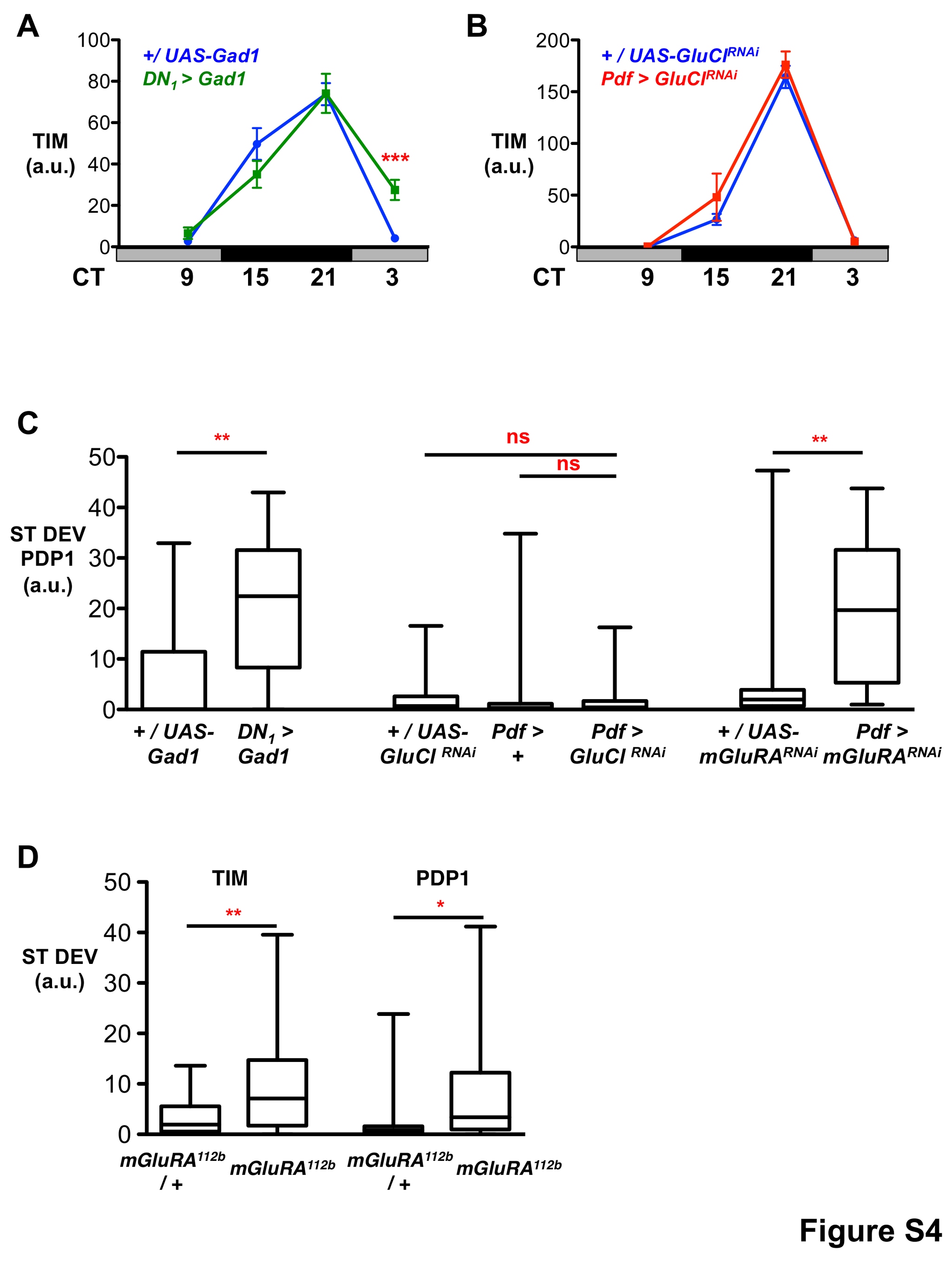

Supplement: Figure S4 — Desynchronization of larval LNvs with altered glutamate signaling. For RNAi experiments, all experimental lines and Pdf>+control lines (Pdf>+) include UAS-Dcr-2. Whiskers represent 95% confidence interval. Error bars represent SEM. * p<0.05; ** p<0.01; *** p<0.001. (A) TIM levels in LNvs were compared between control (UAS-Gad1, blue) and DN1>Gad1 (green) larval brains. Reducing DN1 glutamate signaling through Gad1 misexpression (DN1>Gad1) leaves TIM oscillations intact in LNvs (ANOVA, F3,52 = 19.63, p<0.0001) but increases TIM levels at CT3 (Student's t test, p<0.0001). (B) TIM levels in LNvs were compared between control (+/UAS-GluClRNAi, blue) and Pdf>GluClRNAi (red) larvae. Reducing GluCl levels in LNvs had no effect on TIM oscillations (ANOVA F3,43 = 78.99, p<0.0001) or TIM expression at CT3 (Student's t test, p = 0.34). (C) Box plots showing quantification of desynchrony through measurement of ST DEV in PDP1 expression in larval LNvs in control, DN1>Gad1, Pdf>GluClRNAi, and Pdf>mGluRARNAi larvae at CT3 on day 3 in DD. DN1>Gad1 (Student's t test, p = 0.0035) and Pdf>mGluRARNAi (ANOVA with Tukey's post hoc test, F2,50 = 10.54, p = 0.0002) significantly increase the ST DEV in PDP1 levels, and therefore desynchrony, compared to parental controls, whereas Pdf>GluClRNAi does not (ANOVA with Tukey's post hoc test, F2,39 = 0.11, p = 0.90). (D) Box plots showing quantification of desynchrony through measurement of ST DEV in TIM (left) and PDP1 (right) in mGluRA112b mutants and controls. The ST DEV of TIM (Student's t test, p = 0.0022) and PDP1 (Student's t test, p = 0.013) is significantly increased in mGluRA112b mutants compared to controls (mGluRA112b/+). (TIF) [file pbio.1001959.s004.tif]

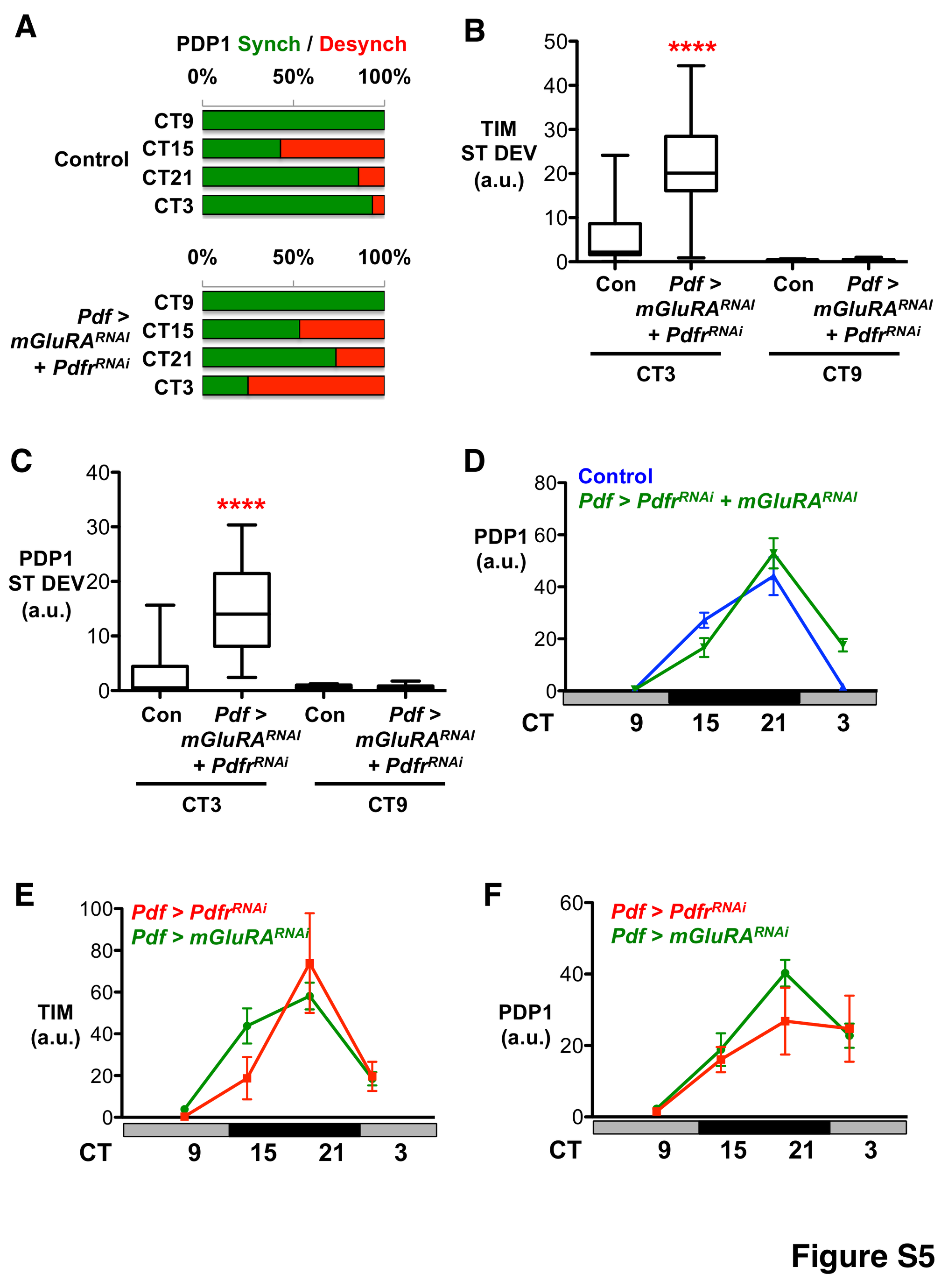

Supplement: Figure S5 — Signaling via mGluRA and PdfR synchronizes LNv clocks. For RNAi experiments, all experimental lines and Pdf>+ control lines include UAS-Dcr-2. Error bars represent SEM. **** p<0.0001. (A) Histogram showing the number of synchronized (green) or desynchronized (red) LNv clusters in control (+/UAS-mGluRARNAI; +/UAS-PdfrRNAi) or Pdf>mGluRARNAi+PdfrRNAi larval brains, determined by PDP1 staining at CT3. (B and C) Box plots quantifying desynchrony by measuring ST DEV in TIM (B) and PDP1 (C) expression in larval LNvs in control (+/UAS-mGluRARNAI; +/UAS-PdfrRNAi) and Pdf>PdfrRNAi+mGluRARNAi larvae at CT3 and CT9 on day 3 in DD. Whiskers represent 95% confidence interval. Pdf>PdfrRNAi+mGluRARNAi significantly increased desynchrony as measured by ST DEV in TIM or PDP1 expression at CT3 but not CT9 compared to controls (ANOVA with Tukey's post hoc test; TIM, F3,47 = 31.96, p<0.0001, and PDP1, F3,47 = 23.43, p<0.0001). (D) Average PDP1 levels of control (blue) and Pdf>mGluRARNAi+PdfrRNAi (green) LNvs. PDP1 oscillates relatively normally in Pdf>mGluRARNAi+PdfrRNAi larval LNvs (two-way ANOVA, no significant genotype effect, F1,82 = 0.15, p = 0.6970). Average TIM (E) and PDP1 (F) levels are shown for Pdf>PdfrRNAi (red) and Pdf>mGluRARNAi (green) LNvs in DD on days 2 and 3. Pdf>mGluRARNAi and Pdf>PdfrRNAi larval LNvs display similar TIM and PDP1 oscillations. TIM, two-way ANOVA, no significant genotype effect (F1,80 = 0.24, p = 0.6224) but a significant time effect (F3,80 = 19.98, p<0.0001). For PDP1, no significant genotype effect (F1,79 = 1.15, p = 0.2876) but a significant time effect (F3,79 = 13.87, p<0.0001). (TIF) [file pbio.1001959.s005.tif]

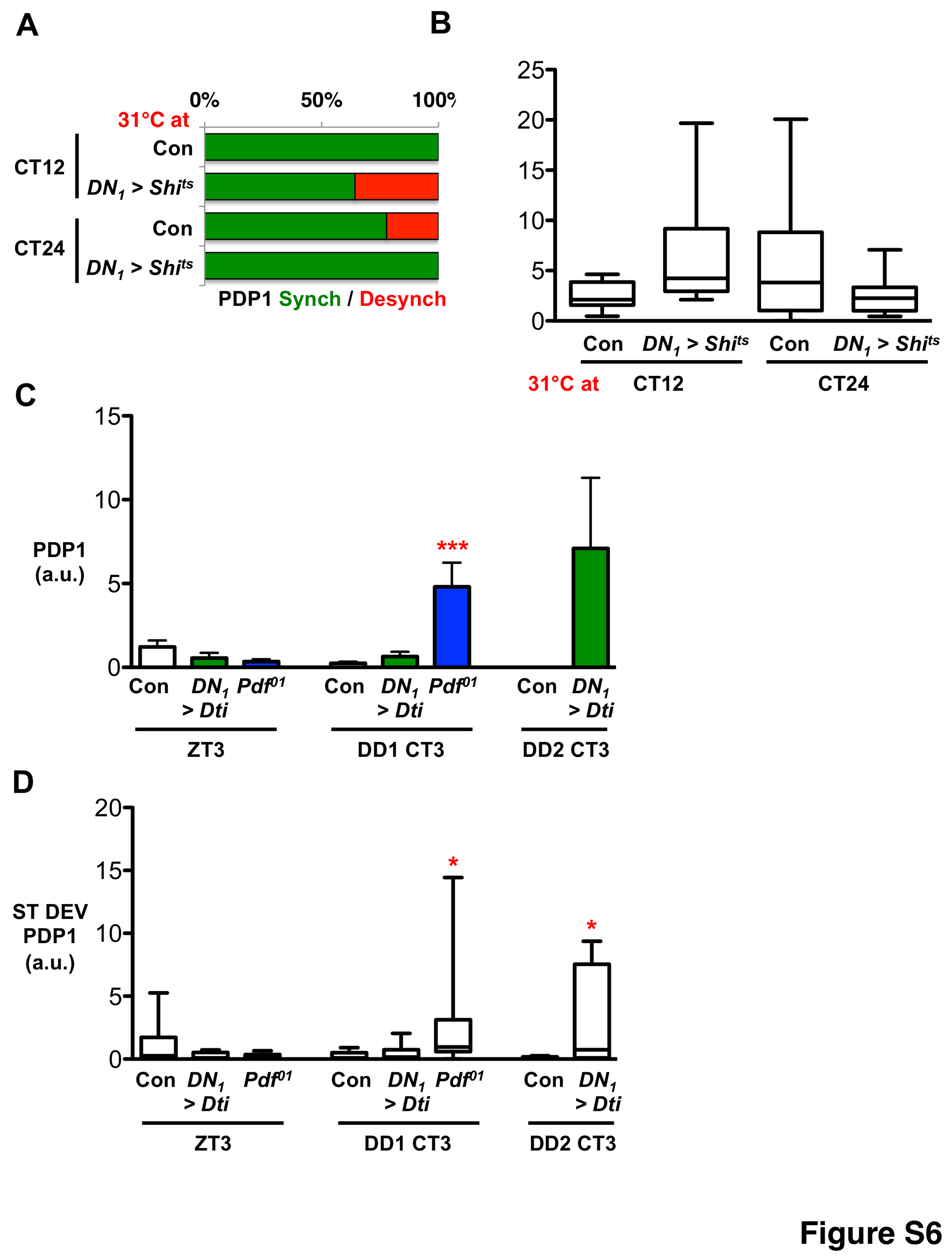

Supplement: Figure S6 — Dawn PDF and Dusk glutamate signals alter LNv PDP1 expression. All statistical comparisons are by ANOVA with Tukey's post hoc test unless otherwise stated. Error bars represent SEM. Whiskers represent 95% confidence interval. * p<0.05; ** p<0.01; *** p<0.005. (A) Histograms showing the percentage of LNv clusters showing synchronized/desynchronized PDP1 expression in control or DN1>shits LNvs after a 6 hour 31°C heat pulse centered at CT12 or CT24. (B) Box plots representing the ST DEV of PDP1 expression in LNvs of control or DN1>shits larvae dissected at CT3 on day 3 of DD after a 31°C heat pulse centered at CT12 or CT24 on day 2 of DD. A heat pulse at CT12 significantly increased the ST DEV in PDP1 expression of DN1>Shits larval LNv clusters (Student's t test, CT12 versus 24, p<0.01), but did not affect controls. (C) Larval LNvs were immunostained for PDP1 at ZT3 and at CT3 on days 1 and 2 of DD in Control (+/UAS-Dti), DN1>Dti, and Pdf01 mutants. DN1 ablation or the Pdf01 mutation do not affect LNv PDP1 levels at ZT 3 (F2,34 = 1.70, p = 0.2). Pdf01 increases TIM expression in LNvs on the first day of DD, whereas DN1>Dti does not (F2,38 = 8.62, p = 0.0008). (D) Desynchrony of LNvs in ZT and on the first and second days of DD was quantified by measuring ST DEV of PDP1 expression in Con (+/UAS-Dti), DN1>Dti, and Pdf01 mutants. There is no difference between genotypes at ZT3 (F2,34 = 2.89, p = 0.07). ST DEV in PDP1 is significantly higher in Pdf01 LNvs compared to control or DN1>Dti LNvs on the first day of DD, reflecting increased desynchrony (F2,38 = 4.62, p = 0.016). DN1>Dti increases desynchrony as measured by PDP1 ST DEV only on day 2 in DD (Student's t test, p = 0.041). (TIF) [file pbio.1001959.s006.tif]

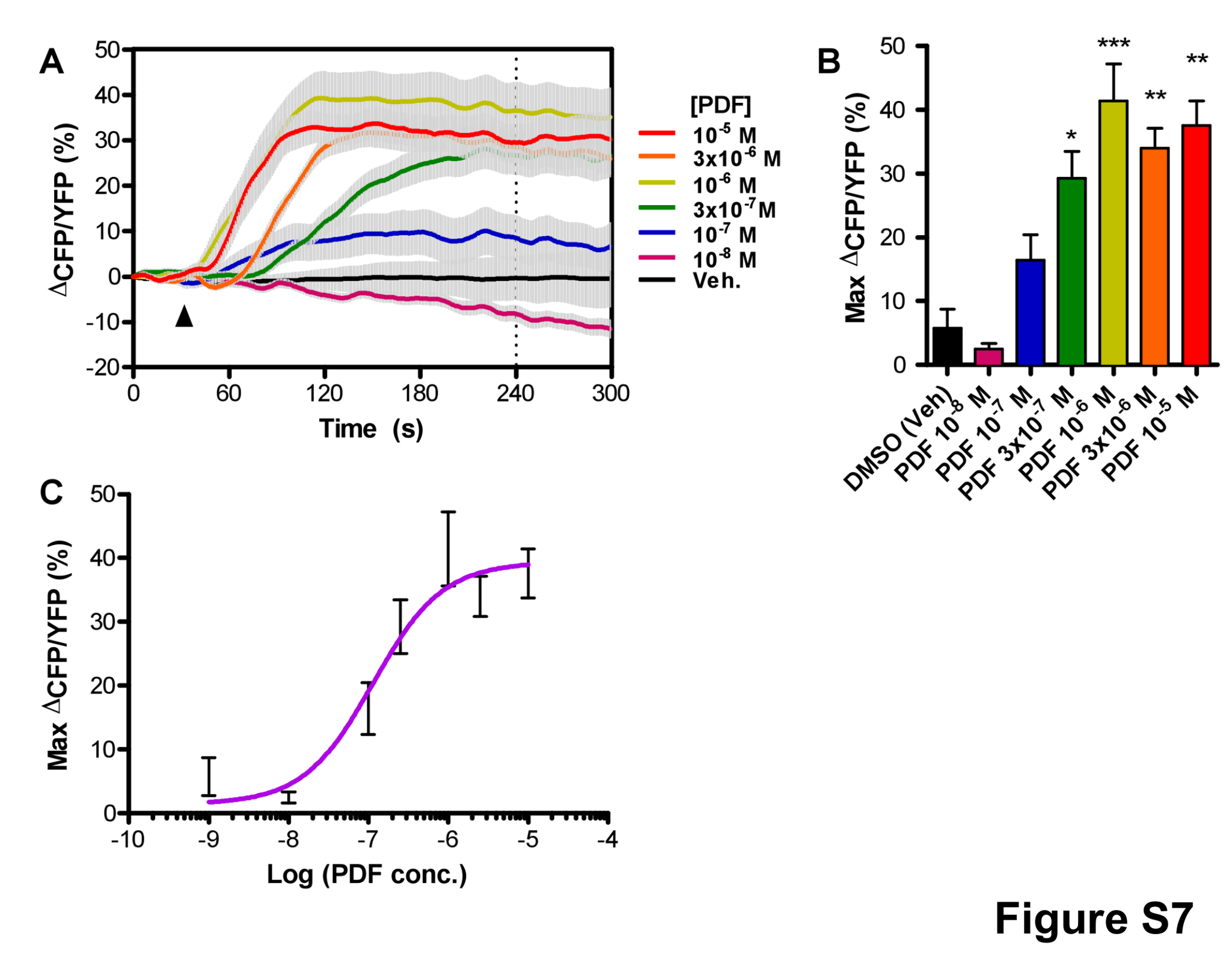

Supplement: Figure S7 — Dose response of larval LNvs to bath-applied PDF. Error bars show SEM. * p<0.05; ** p<0.01; *** p<0.001. (A) Averaged Epac-1-camps CFP/YFP ratio responses to bath application (triangle) of a range of PDF concentrations and vehicle. Sample sizes were as follows: vehicle, seven LNv cell bodies imaged from five brains (7, 5), PDF 10−8 M: (12, 5), PDF 10−7 M: (15, 6), PDF 3×10^−7^ M: (17, 6), PDF 10−6 M: (13, 5), PDF 3×10^−6^ M: (14, 5), and PDF 10−5 M: (16, 6). Error bars represent SEM. (B) Comparison of mean maximum Epac-1-camps CFP/YFP ratio changes between 0 and 240 s (dashed line in A) for the neurons shown in (A). cAMP responses to the various PDF doses were compared by means of a Kruskal–Wallis one-way ANOVA, and a Dunn's multiple comparison test was performed to determine which treatments within the group of compounds tested produced responses significantly different from vehicle controls. (C) Data from (B) fitted as a dose–response curve. The EC50 is 1.1×10^−7^ M PDF. (TIF) [file pbio.1001959.s007.tif]

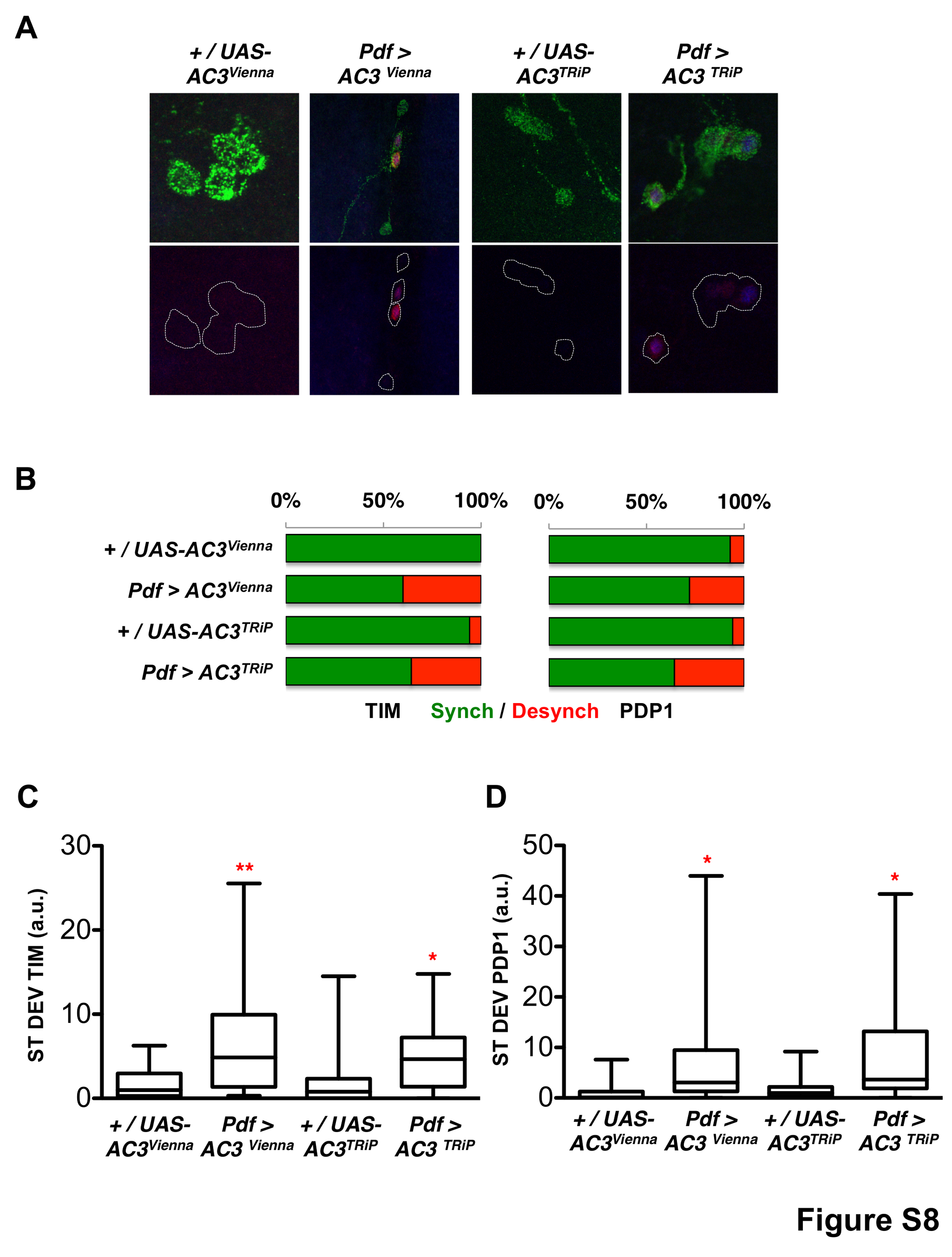

Supplement: Figure S8 — Adenylate cyclase 3 is required in LNvs for synchrony. Desynchrony data are calculated from 3–5 independent experiments, each consisting of at least four brains. Total number of LNv clusters analyzed are in Table S1. Error bars show SEM. Whiskers represent 95% confidence. * p<0.05; ** p<0.01. (A) Representative images of LNvs in control larvae (UAS-RNAi transgene/+) or in larvae with LNvs expressing one of two independent RNAi transgenes targeting AC3 (Pdf>AC3TRiP or Pdf>AC3Vienna) immunostained for PDF (green), TIM (red), and PDP1 (blue) at CT3 on day 3 in DD. The lower panels for each genotype are the same images with the green channel (PDF) removed and replaced by a dashed white line outlining LNvs. (B) Histograms show the percentage of LNv clusters in which TIM (left) or PDP1 (right) was detected in either none or all four of the four LNvs (“synchronized,” green bars) or in one, two, or three LNvs (“desynchronized,” red bars). Box plots showing the ST DEV in (C) TIM or (D) PDP1 expression as in Figure 1. Statistical comparisons show reducing AC3 expression in LNvs via Pdf>AC3TRiP or Pdf>AC3Vienna significantly increases the ST DEV of TIM and PDP1 levels compared to respective controls, reflecting increased desynchrony. (TIF) [file pbio.1001959.s008.tif]

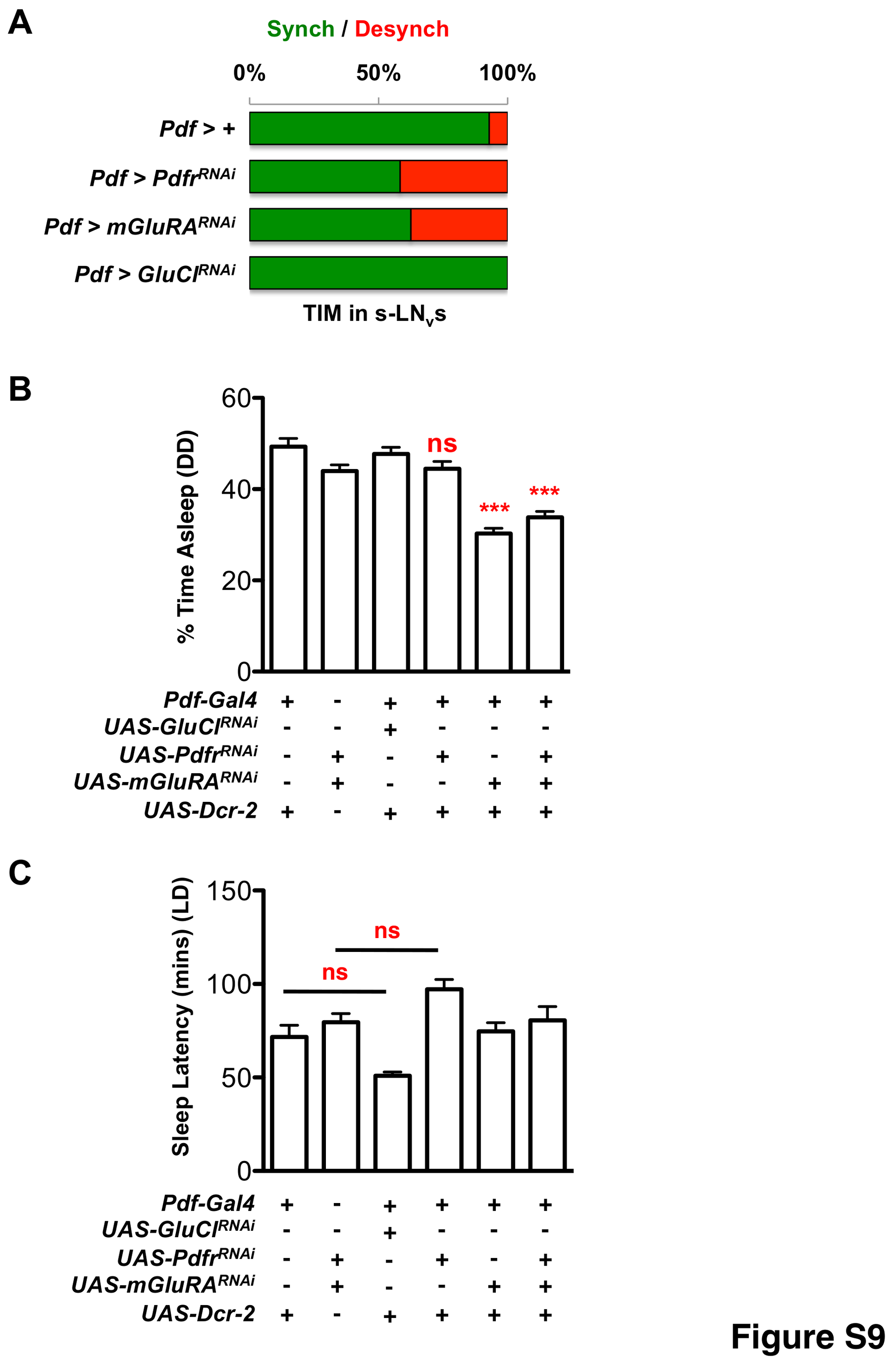

Supplement: Figure S9 — Effects of reduced PdfR and mGluRA signaling in adult LNvs. Error bars represent SEM. Statistics shown represent the least significant difference to all control genotypes as calculated by ANOVA with Tukey's post hoc test. *** p<0.001. (A) Histogram showing the percentage of s-LNv clusters showing synchronized (green) or desynchronized (red) TIM expression in Control (Pdf>+) flies or flies expressing RNAi transgenes targeting mGluRA, Pdfr, or GluCl. (B) Histogram shows the percentage of time spent asleep over the first 5 d in DD. Pdf>mGluRARNAi (ANOVA F = 31.32, p<0.0001) and Pdf>PdfrRNAi+mGluRARNAi (ANOVA F = 23.84, p<0.0001) flies show significantly reduced time spent asleep compared to Pdf>+, +/UAS-mGluRARNAI; +/UAS-PdfrRNAi, or Pdf>GluClRNAi flies. (C) Histogram shows the average sleep latency in LD. By ANOVA, there are no significant differences in sleep latency between Pdf>mGluRARNAi, Pdf>PdfrRNAi, or Pdf>PdfrRNAi+mGluRARNAi flies and UAS-PdfrRNAi+UAS-mGluRARNAi/+ controls. (TIF) [file pbio.1001959.s009.tif]
