# Supplementary material for: Differentially Timed Extracellular Signals Synchronize Pacemaker Neuron Clocks
Source: PLoS Biol. 2014 Sep 30;12(9):e1001959. doi: 10.1371/journal.pbio.1001959 (PMC4181961; doi:10.1371/journal.pbio.1001959)
Supplement: Table S1 — Number of LNvs expressing TIM or PDP1 in each LNv cluster analyzed. Numbers indicate the number of clusters with zero, one, two, three, or four LNvs expressing TIM or PDP1 for each genotype. (PDF) [file pbio.1001959.s010.pdf]

		TIM								PDP1							
**Genotype**	**Time**	**0**	**1**	**2**	**3**	**4**	**% Synch**	**% Deynch**	**n**	**0**	**1**	**2**	**3**	**4**	**% Synch**	**% Deynch**	**n**
y w	CT9	10	0	0	0	0	100	0	10	10	1	0	0	0	90.9	9.1	11
y w	CT15	0	0	0	4	7	63.6	36.4	11	0	0	0	0	11	100.0	0.0	11
y w	CT21	0	0	0	2	14	87.5	12.5	16	0	1	0	2	13	81.3	18.8	16
y w	CT3	20	1	0	0	0	95.2	4.8	21	21	0	0	0	0	100.0	0.0	21
$PdfR^{han}$	CT9	6	0	0	0	0	100	0	6	6	0	0	0	0	100.0	0.0	6
$PdfR^{han}$	CT15	0	0	0	3	8	72.7	27.3	11	0	0	0	2	9	81.8	18.2	11
$PdfR^{han}$	CT21	0	0	0	1	11	91.7	8.3	12	0	0	0	0	12	100.0	0.0	12
$PdfR^{han}$	CT3	15	4	5	6	1	51.6	48.4	31	15	#	3	1	2	54.8	45.2	31
Pdf^{01}	CT9	13	0	0	0	0	100	0	13								
Pdf^{01}	CT15	1	2	5	5	1	14.3	85.7	14								
Pdf^{01}	CT21	0	1	3	3	6	46.2	53.8	13								
Pdf^{01}	CT3	10	5	2	3	0	50	50	20								
Pdf^{01} / +	CT3	13	1	0	0	0	92.9	7.1	14								
y w	ZT3	8	0	0	0	0	100	0	8	8	0	0	0	0	100	0	8
Pdf^{01}	ZT3	8	0	0	0	0	100	0	8	8	0	0	0	0	100	0	8
$PdfR^{han}$	ZT3	7	1	0	0	0	87.5	12.5	8	6	0	0	2	0	75	25	8
$UAS\text{-}PdfR^{RNAi}$ / +	CT3	9	0	0	0	0	100	0	9	8	1	0	0	0	88.9	11.1	9
Pdf > / +	CT3	30	2	0	0	0	93.8	6.3	32	22	4	2	0	2	80	20	30
$Pdf > PdfR^{RNAi}$	CT3	12	3	2	6	2	56	44	25	9	6	2	4	4	52	48	25
$Pdf\text{-}Gal80$ / $UAS\text{-}PdfR^{RNAi}$	CT3	12	4	1	0	1	72.2	27.8	18	13	3	1	1	0	72.2	27.8	18
tim > / +	CT3	14	3	1	0	0	77.8	22.2	18	15	2	1	0	0	83.3	16.7	18
$tim; Pdf\text{-}Gal80 > PdfR^{RNAi}$	CT3	7	3	2	3	3	55.6	44.4	18	8	3	2	2	3	61.1	38.9	18
DN_1 / +	CT3	10	2	0	0	0	83.3	16.7	12	11	1	0	0	0	91.7	8.3	12
UAS-Dti / +	CT3	15	1	0	0	0	93.8	6.3	16	16	0	0	0	0	100	0	16
UAS-Dti / +	CT9	8	0	0	0	0	100	0	8	7	1	0	0	0	87.5	12.5	8
$DN_1 > Dti$	CT3	7	6	2	0	1	50	50	16	9	6	0	0	1	62.5	37.5	16
$DN_1 > Dti$	CT9	10	1	0	0	0	90.9	9.1	11	11	0	0	0	0	100	0	11
UAS-Dti / +	ZT3	9	0	0	0	0	100	0	9	9	0	0	0	0	100	0	9
$DN_1 > Dti$	ZT3	8	0	0	0	0	100	0	8	8	0	0	0	0	100	0	8
UAS-Gad1 / +	CT3	12	0	2	0	0	85.7	14.3	14	11	0	0	3	0	78.6	21.4	14
$DN_1 > Gad1$	CT3	2	3	3	4	2	28.6	71.4	14	3	3	1	5	2	35.7	64.3	14
$Pdf > GluCl^{RNAi}$	CT3	11	3	0	0	0	78.6	21.4	14	13	1	0	0	0	92.9	7.1	14
$UAS\text{-}mGluRA^{RNAi}$ / +	CT3	14	3	0	1	0	77.8	22.2	18	15	3	0	0	0	83.3	16.7	18
$Pdf >mGluRA^{RNAi}$	CT3	8	7	5	1	0	38.1	61.9	21	6	3	9	3	0	28.6	71.4	21
$mGluRA^{112b}$ / +	CT3	23	2	0	0	0	92	8	25	23	1	0	1	0	92	8	25
$mGluRA^{112b}$	CT3	11	5	4	3	1	50	50	24	14	2	1	4	3	70.8	29.2	24
$Pdf > mGluRA^{RNAi} + PdfR^{RNAi}$	CT9	15	0	0	0	0	100	0	15	15	0	0	0	0	100	0	15
$Pdf > mGluRA^{RNAi} + PdfR^{RNAi}$	CT15	9	5	0	1	0	60	40	15	6	4	3	0	2	53.3	46.7	15
$Pdf > mGluRA^{RNAi} + PdfR^{RNAi}$	CT21	4	4	5	1	1	33.3	66.7	15	0	1	1	2	11	73.3	26.7	15
$Pdf > mGluRA^{RNAi} + PdfR^{RNAi}$	CT3	1	7	5	2	1	12.5	87.5	16	3	3	5	4	1	25	75	16
$UAS\text{-}mGluRA^{RNAi} + UAS\text{-}PdfR^{RNAi}$ / +	CT9	6	0	0	0	0	100	0	6	6	0	0	0	0	100	0	6
$UAS\text{-}mGluRA^{RNAi} + UAS\text{-}PdfR^{RNAi}$ / +	CT15	0	0	3	5	6	42.9	57.1	14	2	2	2	4	4	42.9	57.1	14
$UAS\text{-}mGluRA^{RNAi} + UAS\text{-}PdfR^{RNAi}$ / +	CT21	0	0	0	0	7	100	0	7	0	1	0	1	5	71.4	28.6	7
$UAS\text{-}mGluRA^{RNAi} + UAS\text{-}PdfR^{RNAi}$ / +	CT3	12	2	1	0	0	80	20	15	14	1	0	0	0	93.3	6.7	15
$DN_1 > Shi^{ts}$ 31C at CT12	CT3	5	4	4	0	1	42.9	57.1	14	9	3	0	2	0	64.3	35.7	14
$DN_1 > Shi^{ts}$ 31C at CT24	CT3	18	0	0	0	0	100	0	18	18	0	0	0	0	100	0	18
$UAS\text{-}Shi^{ts}$ 31C at CT12	CT3	13	1	0	0	0	92.9	7.1	14	14	0	0	0	0	100	0	14
$UAS\text{-}Shi^{ts}$ 31C at CT24	CT3	16	2	0	0	0	88.9	11.1	18	14	2	1	1	0	77.8	22.2	18
$Pdf > AC3^{TRiP}$	CT3	9	4	1	0	0	64.3	35.7	14	9	4	1	0	0	64.3	35.7	14
$UAS\text{-}AC3^{TRiP}$ / +	CT3	16	0	1	0	0	94.1	5.9	17	16	1	0	0	0	94.1	5.9	17
$Pdf > AC3^{Vienna}$	CT3	15	9	1	0	0	60	40	25	18	7	0	0	0	72	28	25
$UAS\text{-}AC3^{Vienna}$	CT3	13	0	0	0	0	100	0	13	13	1	0	0	0	92.9	7.1	14
Pdf > UAS-AC3	CT3	7	5	0	1	0	53.8	46.2	13	9	3	1	0	0	69.2	30.8	13
UAS-AC3 / +	CT3	13	0	0	0	0	100	0	13	13	0	0	0	0	100	0	13
Adult:																	
$UAS\text{-}mGluRA^{RNAi} + UAS\text{-}PdfR^{RNAi}$ / +	CT9	6	1	0	0	0	85.7	14.3	7								
$UAS\text{-}mGluRA^{RNAi} + UAS\text{-}PdfR^{RNAi}$ / +	CT15	2	1	0	0	9	91.7	8.3	12								
$UAS\text{-}mGluRA^{RNAi} + UAS\text{-}PdfR^{RNAi}$ / +	CT21	0	0	0	0	12	100	0	12								
$UAS\text{-}mGluRA^{RNAi} + UAS\text{-}PdfR^{RNAi}$ / +	CT3	7	1	1	0	2	81.8	18.2	11								
$Pdf > mGluRA^{RNAi} + PdfR^{RNAi}$	CT9	10	2	0	0	0	83.3	16.7	12								
$Pdf > mGluRA^{RNAi} + PdfR^{RNAi}$	CT15	4	2	2	3	1	41.7	58.3	12								
$Pdf > mGluRA^{RNAi} + PdfR^{RNAi}$	CT21	0	1	0	8	4	30.8	69.2	13								
$Pdf > mGluRA^{RNAi} + PdfR^{RNAi}$	CT3	3	3	1	2	2	45.5	54.5	11								
