# Supplementary material for: Differentially Timed Extracellular Signals Synchronize Pacemaker Neuron Clocks
Source: PLoS Biol. 2014 Sep 30;12(9):e1001959. doi: 10.1371/journal.pbio.1001959 (PMC4181961; doi:10.1371/journal.pbio.1001959)
Supplement: Table S2 — Behavioral periods and strengths of behavioral rhythms in adult flies with altered glutamate and PDF receptor expression in LNvs. (PDF) [file pbio.1001959.s011.pdf]

Genotype	Period	SEM	Power	SEM	n
Pdf > / +	24.21	0.04	696.3	43.7	41
Pdf > GluCl^{RNAi}	24.30	0.05	868	53.4	40
Pdf > mGluRA^{RNAi}	24.55	0.04	559	36.4	44
Pdf > PdfR^{RNAi}	24.33	0.07	468	56.6	23
Pdf > PdfR^{RNAi} + mGluRA^{RNAi}	24.57	0.04	625.6	55	29
UAS-Pdfr^{RNAi} + UAS-mGluRA^{RNAi} / +	24.09	0.06	376.5	52	18
